# The association between triglyceride glucose-waist height ratio index and cardiometabolic multimorbidity among Chinese middle-aged and older adults: a national prospective cohort study

**DOI:** 10.1186/s12933-025-02919-x

**Published:** 2025-09-02

**Authors:** Longyan Lv, Ping Zhang, Xuerui Chen, Yan Gao

**Affiliations:** 1 First Clinical Medical College, Shandong University of Traditional Chinese Medicine, Jinan, Shandong Province 250355 China; 2 Department of General Medicine, the 960th Hospital of People’s Liberation Army Joint Logistics Support Force, NO.25 Shifan Road, Jinan, Shandong 250031 People’s Republic of China

**Keywords:** Triglyceride glucose-waist height ratio index (TyG-WHtR), Cardiometabolic multimorbidity (CMM), CHARLS

## Abstract

**Background:**

Cardiometabolic multimorbidity (CMM) imposes a progressively severe health burden worldwide. Triglyceride-glucose (TyG) index and waist-to-height ratio (WHtR), as indicators of insulin resistance and central adiposity, respectively, have been shown to be strongly associated with CMM. However, there is currently a lack of research combining the two for CMM risk assessment. This study aims to investigate the relationship between TyG-WHtR index and CMM.

**Methods:**

This prospective cohort study analyzed data from Chinese adults aged ≥ 45 years participating in the 2011–2020 waves of the China Health and Retirement Longitudinal Study (CHARLS). We employed the Kaplan-Meier curves, multivariable Cox regression analysis, and restricted cubic spline (RCS) to examine the relationship between the TyG-WHtR index and the risk of CMM. Time-dependent receiver operating characteristic (ROC), net reclassification improvement (NRI), and integrated discrimination improvement (IDI) analyses were utilized to evaluate predictive performance. Additionally, subgroup analyses and sensitivity tests were conducted to assess the robustness of the findings.

**Results:**

During a median follow-up of 9 years, 413 (9.4%) of the 4393 participants developed CMM. Multivariable Cox regression analysis revealed progressively higher risks of CMM across increasing TyG-WHtR quartiles. Compared to participants in the lowest quartile (Q1) of the TyG-WHtR index, the hazard ratios (HRs) and 95% confidence intervals (CIs) for those in quartiles Q2, Q3, and Q4 were 1.75 (1.18–2.6), 2.33 (1.58–3.43), and 3.13 (2.08–4.7), respectively. Consistently, elevated cumulative TyG-WHtR independently increased CMM risk. The RCS analysis indicated a positive linear relationship between the TyG-WHtR index and the incidence of CMM. Moreover, both baseline and cumulative TyG-WHtR significantly improved reclassification metrics (NRI/IDI) and discriminative ability (AUC). Sensitivity analyses corroborated these primary findings.

**Conclusion:**

This study suggests that TyG-WHtR independently predicts CMM risk. The linear dose-response relationship highlight the potential utility of TyG-WHtR in early risk assessment and prevention strategies for CMM.

**Graphical abstract:**

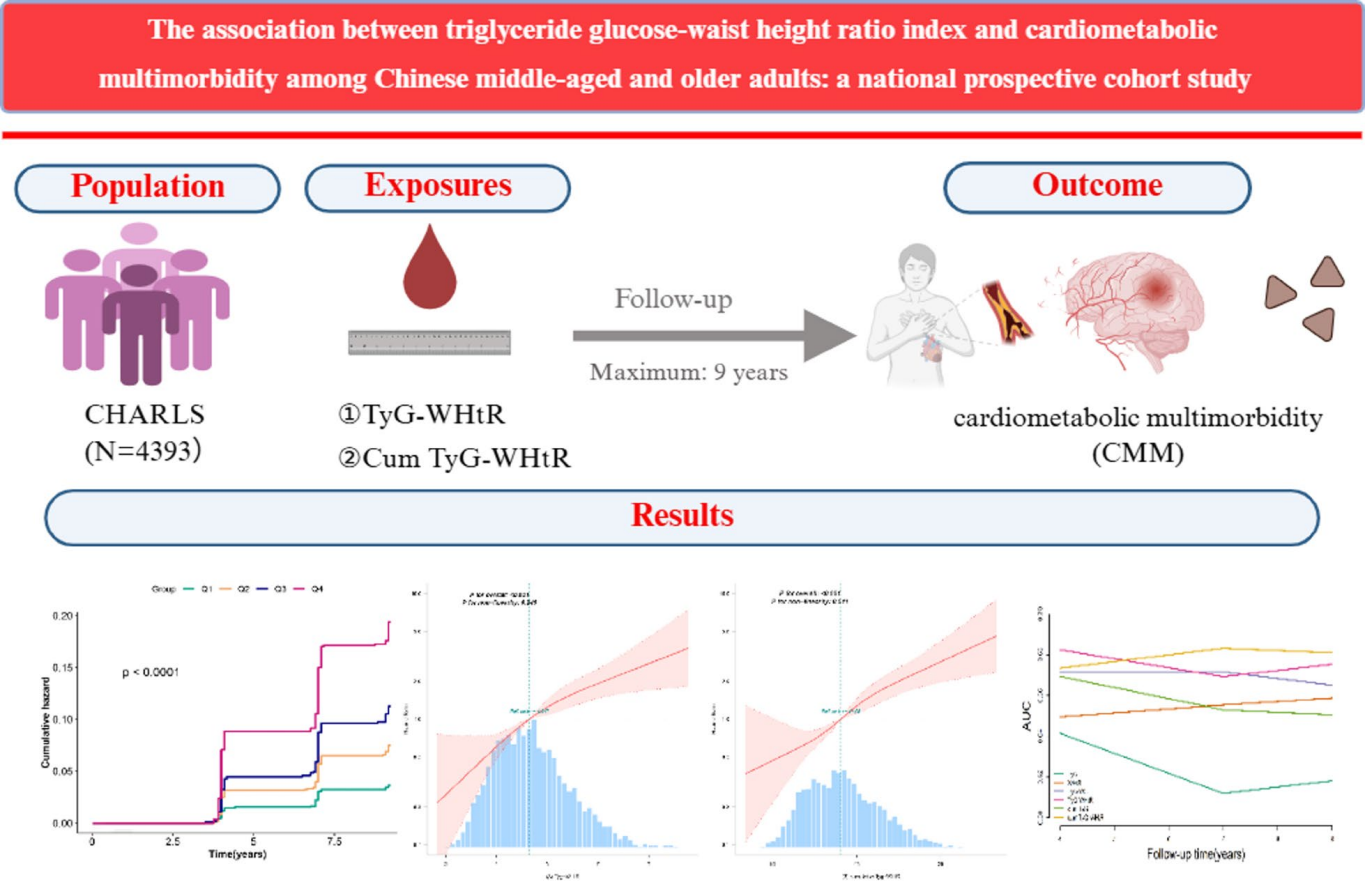

**Supplementary Information:**

The online version contains supplementary material available at 10.1186/s12933-025-02919-x.

## Research insights

**What is currently known about this topic?**

- Cardiometabolic multimorbidity (CMM) poses a severe global health burden, and while the TyG index (insulin resistance) and WHtR (central adiposity) are individually associated with CMM, research combining them for risk prediction is lacking.

**What is the key research question?**

- Can the TyG-WHtR index predict incident CMM in middle-aged and older Chinese adults?

**What is new?**

- This study shows that the TyG-WHtR index is a novel predictor of CMM risk, with a positive linear relationship between the index and CMM incidence. Elevated cumulative TyG-WHtR levels are independently associated with increased CMM risk.

**How might this study influence clinical practice?**

- The TyG-WHtR index could be a valuable tool for early CMM risk assessment, enabling targeted prevention strategies and personalized interventions to reduce CMM incidence.

## Introduction

Multimorbidity, characterized by the presence of two or more chronic conditions in an individual, poses a significant threat to human health, especially among middle-aged and older adults [1]. Cardiometabolic multimorbidity (CMM) is a particularly salient and clinically consequential manifestation of multimorbidity [2, 3]. CMM is specifically defined as the concurrent presence of a minimum of two cardiometabolic diseases (CMDs), typically including heart disease, diabetes, and stroke [4]. Epidemiological studies report a CMM prevalence rate of 5.94–16.9% in China, with this alarming disease burden posing a major public health challenge [5, 6]. Notably, CMM prevalence is projected to rise against the backdrop of a rapidly aging population and escalating global obesity rates [7]. Multiple studies have confirmed that patients with CMM face significantly higher cumulative health risks compared to those with a single CMD or none, including increased all-cause mortality [8], cognitive decline [9, 10], and decreased quality of life [11]. Current research predominantly focuses on individual CMDs, while investigations into CMM remain scarce. Given its escalating prevalence and associated health hazards, early detection and targeted interventions for CMM are imperative to mitigate adverse clinical outcomes [12].

Insulin resistance (IR) is defined as a condition in which the body exhibits impaired insulin-mediated regulation of glucose metabolism in target tissues [13]. Mediated by multiple factors, IR significantly contributes to the onset and progression of cardiovascular and metabolic diseases [14]. Current methodologies utilized for evaluating IR, such as the hyperinsulinemic-euglycemic clamp (gold standard) and HOMA-IR, are technically complex and lack clinical accessibility [15]. In this context, the TyG index has garnered recognition as a viable and economical surrogate biomarker for evaluating IR [16]. Studies have shown that it is correlated with various cardiometabolic diseases, including adverse cardiovascular event [17], ischemic stroke [18], and prediabetes [19].

The global obesity epidemic, characterized by IR and dysregulated lipid metabolism, substantially increases risks of diabetes and cardiovascular disease [20]. Central obesity, a primary driver of obesity-associated cardiometabolic disorders, is largely attributable to visceral adipose tissue (VAT) accumulation [21]. Standard anthropometric proxies for VAT include waist circumference (WC), waist-to-hip ratio (WHR), and waist-to-height ratio (WHtR). However, WC cannot distinguish VAT from subcutaneous fat, while WHR measurements exhibit high variability due to inconsistent hip circumference assessment protocols [22]. In contrast, WHtR provides a standardized approach for evaluating central obesity through its height-normalized metric [23]. Notably, TyG combining with adiposity indices demonstrates superior predictive capacity for IR and cardiometabolic risk stratification compared to using TyG alone [24]. While extant studies have shown that TyG-WHtR is associated with cardiovascular disease [25, 26], investigations to date have centered predominantly on single CMDs.

Given the paucity of population-based evidence on the TyG-WHtR index as a predictor of CMM, this nationwide study utilized data from the China Health and Retirement Longitudinal Study (CHARLS) to analyze the association in a representative sample of Chinese adults aged 45 years and older.

## Methods

### Data source and study population

The participants of this study were middle-aged and older adults enrolled in the CHARLS, a prospective national cohort study conducted in China. The CHARLS initiated its national baseline survey (Wave 1) during June 2011-March 2012, enrolling 17,708 participants across 28 provinces. Participants underwent biennial follow-ups through 2013–2020: Wave 2 (2013–2014), Wave 3 (2015–2016), Wave 4 (2017–2018), and Wave 5 (2019–2020). A comprehensive account of the study’s design and the methodology of data collection for the CHARLS has been previously documented [27]. The data set is accessible for download at http://CHARLS.pku.edu.cn/en. The CHARLS survey was conducted in accordance with the Declaration of Helsinki and had received approval from the BU Institutional Review Board (IRB 00001052–11015). Informed consent was obtained in writing from all participants prior to their enrollment in the study. CHARLS field staff received rigorous professional training and used a standardized questionnaire during face-to-face interviews. This study defined the 2011–2012 cohort as baseline, with longitudinal follow-up at three timepoints: 2015, 2018, and 2020.

**Fig. 1:**
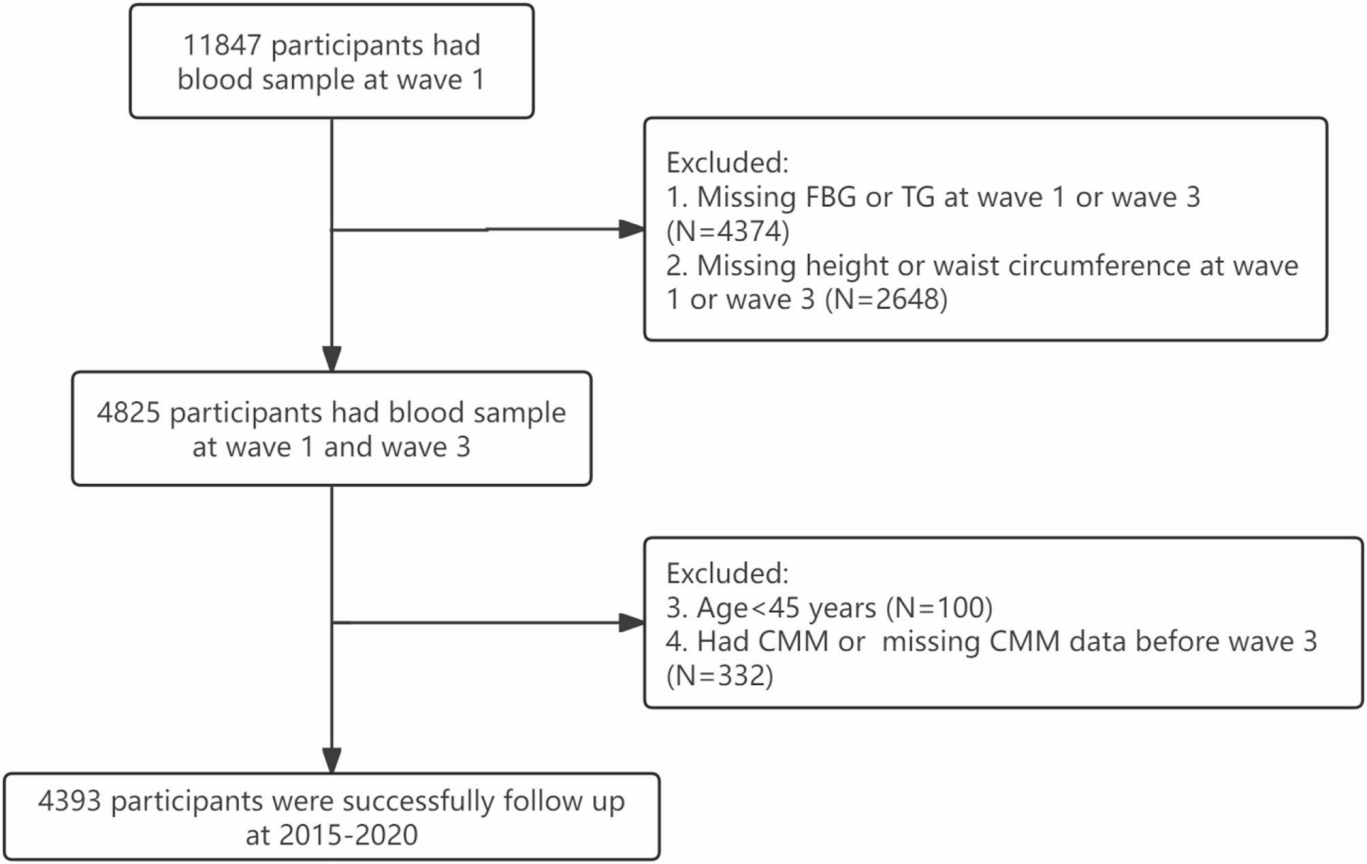
Flow chart of inclusion and exclusion criteria of participants

This study initially enrolled 11,847 individuals undergoing blood collection. First, we excluded participants with missing data on triglycerides (TG), fasting blood glucose (FBG), height, or waist measurements at Wave 1 or Wave 3. Next, we excluded participants younger than 45 years old. Then, we excluded individuals who had CMM or lacked CMM-related information before Wave 3 (2015). Ultimately, the study included a total of 4393 participants (Fig. 1).

### Assessment of TyG-WHtR

The following formulas were used to calculate TyG-WHtR and cumulative TyG-WHtR [25, 28]:

$$ \begin{aligned} & {\text{TyG}} = \ln \left[ {\frac{{{\text{triglycerides~}}\left( {{\text{mg}}/{\text{dl}}} \right) \times {\text{glucose~}}\left( {{\text{mg}}/{\text{dl}}} \right)}}{2}} \right] \\ & {\text{WHtR}} = \frac{{{\text{waist}}\,{\text{circumference}}}}{{{\text{height}}}} \\ & {\text{TyG}} - {\text{WHtR}} = {\text{TyG}} \times {\text{WHtR}} \\ & {\text{cumulative~}}\,{\text{TyG}} - {\text{WHtR}} = \frac{{\left( {{\text{TyG}} - {\text{WHtR}}2012 + {\text{TyG}} - {\text{WHtR}}2015} \right)}}{2} \\ & \,\,\,\,\,\,\,\,\,\,\,\,\,\,\,\,\,\,\,\,\,\,\,\,\,\,\,\,\,\,\,\,\,\,\,\,\,\,\,\,\,\,\,\,\,\,\,\,\,\,\,\,\,\,\,\,\,\,\,\,\,\,\,\,\,\,\quad \times {\text{time}}\left( {2015 - 2012} \right) \\ \end{aligned} $$

### Assessment of CMM

The primary endpoint of this study was the occurrence of CMM events, characterized by the simultaneous presence of at least two CMDs, including heart disease, stroke, and diabetes [29]. The diagnosis of heart disease and stroke is based on self-reported data or related medication use collected at baseline and follow-up surveys. A diagnosis of diabetes was made if any of the following were met: (1) self-reported diagnosis of diabetes (“Have you been diagnosed with diabetes or hyperglycemia by your doctor?”); (2) current use of glucose-lowering medications; (3) FBG ≥ 126 mg/dL; or (4) hemoglobin A1c (HbA1c) ≥ 6.5%. For secondary outcomes, we performed further analyses for diabetes, heart disease, and stroke, respectively. Our CMM diagnostic criteria are consistent with those used in previous literature based on CHARLS data [30].

### Covariates

In this study, the following five main components were included as covariates: (1) Demographic data: baseline age (years, continue variable), sex (female and male), marital status (married and others), educational level(illiteracy, middle school below and middle school and above), residence (rural village and urban community). (2) Lifestyle data: smoking status (never and others), Drinking status (current and others). (3) Disease history (yes or no): hypertension, dyslipidemia, cancer. (4) Body measures: systolic blood pressure (SBP), diastolic blood pressure (DBP). (5) Laboratory examination data: total cholesterol (TC), high-density lipoprotein cholesterol (HDL-C), and low-density lipoprotein cholesterol (LDL-C)). Hypertension was identified based on SBP ≥ 140 mmHg or DBP ≥ 90 mmHg or a history of hypertension, or the use of antihypertensive medications [31]. Dyslipidemia was determined by self-reported dyslipidemia or the use of lipid-lowering therapies.

### Handling of missing data and data preprocessing

The degree of missing data in this study is detailed in Table S1. Before formal analyses, multicollinearity assessment was performed for all study variables. Variables with significant collinearity were defined as GVIF^(1/2DF) ≥ 2, where GVIF denotes generalized variance inflation factor and DF indicates degrees of freedom (Table S2). Additionally, to reduce bias resulting from missing variables, we used multiple imputation by chained equations (MICE) to address the missing data.

During data preprocessing, extreme values were detected in TyG and WHtR indices. A percentile-based Winsorization method was implemented to mitigate outlier effects and improve analytical robustness. Values beyond the 1st and 99th percentiles were replaced with corresponding thresholds, retaining the central 98% distribution.

### Statistical analyses

For normally distributed data, continuous variables were expressed as mean ± standard deviation (SD). For skewed data, continuous variables were reported as the median and interquartile range. Categorical variables were presented as counts with corresponding percentages. Based on the quartiles of TyG-WHtR and cumulative TyG-WHtR, we divided the participants into four groups: Q1, Q2, Q3, and Q4. Kaplan-Meier survival curves were utilized to analyze the cumulative hazard of CMM based on the TyG-WHtR quartile. Multivariable-adjusted Cox regression models were employed to estimate hazard ratios (HRs) and 95% confidence levels (CIs) for the association of TyG-WHtR (including baseline levels and cumulative exposure) with CMM risk. Four models with progressive adjustments were constructed: Model 1 adjusted for age and sex; Model 2 additionally for sociodemographic factors (marital status, residence, education level, smoking status, and drinking status.); Model 3 further for comorbidities (hypertension, dyslipidemia, cancer history); and Model 4 additionally for clinical biomarkers (SBP, DBP, TC, HDL-C, LDL-C). Additionally, to further explore the dose-response relationship between TyG-WHtR and CMM, we performed a restricted cubic spline (RCS) analysis with four nodes (5th, 35th, 65th, and 95th) and adjusted for the variables in Model 4. Time-dependent receiver operating characteristic (ROC) curves were used to quantify the discriminative accuracy of the TyG-WHtR index for incident CMM during follow-up. Net reclassification index (NRI) and integrated discrimination improvement (IDI) index were also employed to further assess the incremental predictive utility compared to the basic models. To verify the robustness of the study findings, the following sensitivity analyses were performed: (1) Repeating the primary analyses using the complete dataset without multiple imputation. (2) Participants with diabetes, heart disease, or stroke at baseline were excluded. (3) To minimize residual confounding, participants with baseline hypertension, dyslipidemia or cancer were excluded. (4) To address potential bias from competing mortality risks, associations were reassessed using Fine-Gray subdistribution hazards regression, treating non-CMM-related deaths as competing events (Additional file: Tables S3, S4 and S5).

All statistical analyses were conducted using R Statistical Software (Version 4.2.2, http://www.R-project.org, The R Foundation) and Free Statistics Analysis Platform (Version 2.1.1, Beijing, China, http://www.clinicalscientists.cn/freestatis tics). Statistical significance was defined as a two-sided *p*-value < 0.05.

## Results

### Baseline characteristics of participants

In this study, a total of 4393 participants with no CMM were included for analysis. Table 1 details baseline characteristics of the study cohort. The mean age (SD) of the participants was 58.76 ± 8.70 years, and 45.8% of them were males. In 2012, the mean TyG-WHtR was 4.71 ± 0.76, and in 2015, it was 4.79 ± 0.81. The mean cumulative TyG-WHtR was 14.26 ± 2.21. Compared to the Q1 group, individuals in Q2-4 were more likely to be illiterate, female, and living in urban areas. They also had higher levels of certain physiological indicators, including SBP, DBP, LDL-C, TC, HbA1C, FBG, WC, height and TG. Additionally, Q2-4 groups had higher proportions of non-smokers and non-drinkers, as well as a higher prevalence of hypertension and dyslipidemia. However, there were no statistically significant disparities observed in age, marital status, or cancer prevalence across the TyG-WHtR groups. Furthermore, we stratified and presented baseline characteristics according to cumulative TyG-WHtR quartiles (Additional file 1: Table S3).

**Table 1:** The baseline characteristics of participants

Characteristic	Total	TyG-WHtR				*P*
	(*n* = 4393)	Q1 (*n* = 1098)	Q2 (*n* = 1098)	Q3 (*n* = 1098)	Q4 (*n* = 1099)	
Age, years (Mean ± SD)	58.76 ± 8.70	59.09 ± 9.17	58.64 ± 8.62	58.32 ± 8.40	59.00 ± 8.56	0.147
Gender, n (%)						< 0.001
Male	2011 (45.8)	707 (64.4)	548 (49.9)	457 (41.6)	299 (27.2)	
Female	2382 (54.2)	391 (35.6)	550 (50.1)	641 (58.4)	800 (72.8)	
Marital status, n (%)						0.564
Married	3933 (89.5)	978 (89.1)	988 (90)	992 (90.3)	975 (88.7)	
Others	460 (10.5)	120 (10.9)	110 (10)	106 (9.7)	124 (11.3)	
Educational level, n (%)						0.007
Illiteracy	1177 (26.8)	268 (24.4)	285 (26)	282 (25.7)	342 (31.1)	
Middle school below	1910 (43.5)	493 (44.9)	478 (43.5)	471 (42.9)	468 (42.6)	
Middle school and above	1306 (29.7)	337 (30.7)	335 (30.5)	345 (31.4)	289 (26.3)	
Residence, n (%)						< 0.001
Rural	3813 (86.8)	1013 (92.3)	957 (87.2)	923 (84.1)	920 (83.7)	
Urban	580 (13.2)	85 (7.7)	141 (12.8)	175 (15.9)	179 (16.3)	
Smoking status, n (%)						< 0.001
Never	2705 (61.6)	496 (45.2)	655 (59.7)	719 (65.5)	835 (76)	
Others	1688 (38.4)	602 (54.8)	443c (40.3)	379 (34.5)	264 (24)	
Drinking status, n (%)						< 0.001
Yes	1402 (31.9)	455 (41.4)	376 (34.2)	322 (29.3)	249 (22.7)	
No	2991 (68.1)	643 (58.6)	722 (65.8)	776 (70.7)	850 (77.3)	
Comorbidities, n (%)						
Hypertension	1023 (23.3)	135 (12.3)	199 (18.1)	281 (25.6)	408 (37.1)	< 0.001
Dyslipidemia	387 (8.8)	41 (3.7)	76 (6.9)	93 (8.5)	177 (16.1)	< 0.001
Cancer	36 (0.8)	7 (0.6)	6 (0.5)	8 (0.7)	15 (1.4)	0.051
SBP, mmHg (Mean ± SD)	130.53 ± 23.65	124.63 ± 22.28	128.01 ± 20.42	130.85 ± 20.55	138.62 ± 28.22	< 0.001
DBP, mmHg (Mean ± SD)	76.16 ± 12.21	72.61 ± 11.54	74.84 ± 11.87	76.82 ± 11.73	80.37 ± 12.36	< 0.001
HDL-C, mg/dL (Mean ± SD)	51.18 ± 15.62	59.37 ± 16.10	54.16 ± 14.52	48.77 ± 13.41	42.42 ± 12.97	< 0.001
LDL-C, mg/dL (Mean ± SD)	116.24 ± 35.46	109.80 ± 30.70	116.75 ± 32.47	121.75 ± 34.02	116.68 ± 42.52	< 0.001
TC, mg/dL (Mean ± SD)	193.41 ± 39.20	182.05 ± 35.34	189.77 ± 36.46	195.70 ± 35.90	206.11 ± 44.42	< 0.001
HbA1C, %	5.23 ± 0.75	5.09 ± 0.55	5.12 ± 0.58	5.22 ± 0.67	5.50 ± 1.03	< 0.001
TG2012, mg/dL	105.3 (74.3, 153.1)	70.8 (55.8, 89.4)	93.8 (71.7, 126.6)	117.7 (90.3, 158.4)	175.2 (124.8, 259.7)	< 0.001
TG2015, mg/dL	115.0 (83.2, 168.1)	85.0 (68.1, 113.3)	104.4 (78.8, 146.0)	124.8 (92.9, 176.1)	167.3 (120.8, 241.6)	< 0.001
FBG2012, mg/dL	109.26 ± 34.10	99.26 ± 17.25	104.62 ± 25.67	108.23 ± 30.97	124.91 ± 48.68	< 0.001
FBG2015, mg/dL	102.58 ± 32.69	94.84 ± 20.95	98.04 ± 24.32	102.57 ± 32.59	114.88 ± 44.19	< 0.001
Height2012 (Mean ± SD)	157.69 ± 8.35	160.07 ± 7.92	157.75 ± 8.14	157.51 ± 8.66	155.44 ± 8.01	< 0.001
WC2012 (Mean ± SD)	85.24 ± 9.95	75.04 ± 5.71	81.76 ± 5.62	88.20 ± 6.13	95.96 ± 7.40	< 0.001
Height 2015 (Mean ± SD)	157.74 ± 23.45	159.50 ± 8.05	157.24 ± 8.24	158.52 ± 36.76	155.71 ± 26.61	0.001
WC2015 (Mean ± SD)	86.26 ± 17.29	77.22 ± 7.37	83.01 ± 7.84	89.20 ± 28.45	95.60 ± 9.14	< 0.001
TyG2012 (Mean ± SD)	8.68 ± 0.67	8.16 ± 0.41	8.50 ± 0.44	8.76 ± 0.48	9.31 ± 0.70	< 0.001
TyG2015 (Mean ± SD)	8.71 ± 0.63	8.33 ± 0.47	8.57 ± 0.52	8.79 ± 0.57	9.16 ± 0.64	< 0.001
WHtR2012 (Mean ± SD)	0.54 ± 0.07	0.47 ± 0.03	0.52 ± 0.03	0.56 ± 0.03	0.62 ± 0.05	< 0.001
WHtR2015 (Mean ± SD)	0.55 ± 0.07	0.48 ± 0.04	0.53 ± 0.05	0.56 ± 0.05	0.62 ± 0.06	< 0.001
TyG-WHtR2012 (Mean ± SD)	4.71 ± 0.76	3.82 ± 0.24	4.39 ± 0.14	4.90 ± 0.16	5.74 ± 0.47	< 0.001
TyG-WHtR2015 (Mean ± SD)	4.79 ± 0.81	4.04 ± 0.48	4.53 ± 0.50	4.95 ± 0.55	5.65 ± 0.67	< 0.001
Heart disease, n (%)	1257 (28.6)	240 (21.9)	282 (25.7)	347 (31.6)	388 (35.3)	< 0.001
Diabetes, n (%)	1169 (26.6)	159 (14.5)	221 (20.1)	299 (27.2)	490 (44.6)	< 0.001
Stroke, n (%)	461 (10.5)	77 (7)	103 (9.4)	125 (11.4)	156 (14.2)	< 0.001
CMM, n (%)	413 (9.4)	38 (3.5)	76 (6.9)	113 (10.3)	186 (16.9)	< 0.001

SBP, systolic blood pressure; DBP, diastolic blood pressure; TC, total cholesterol; TG, triglycerides; HDL-C, high-density lipoprotein cholesterol; LDL-C, low-density lipoprotein cholesterol; HbA1C, glycated hemoglobin; FBG, fasting blood glucose; WC, waist measurements; TyG, triglyceride-glucose; WHtR, waist height ratio; TyG-WHtR, triglyceride glucose-waist height ratio index; Q, quartile

### Associations between TyG-WHtR and incident CMM

**Fig. 2:**
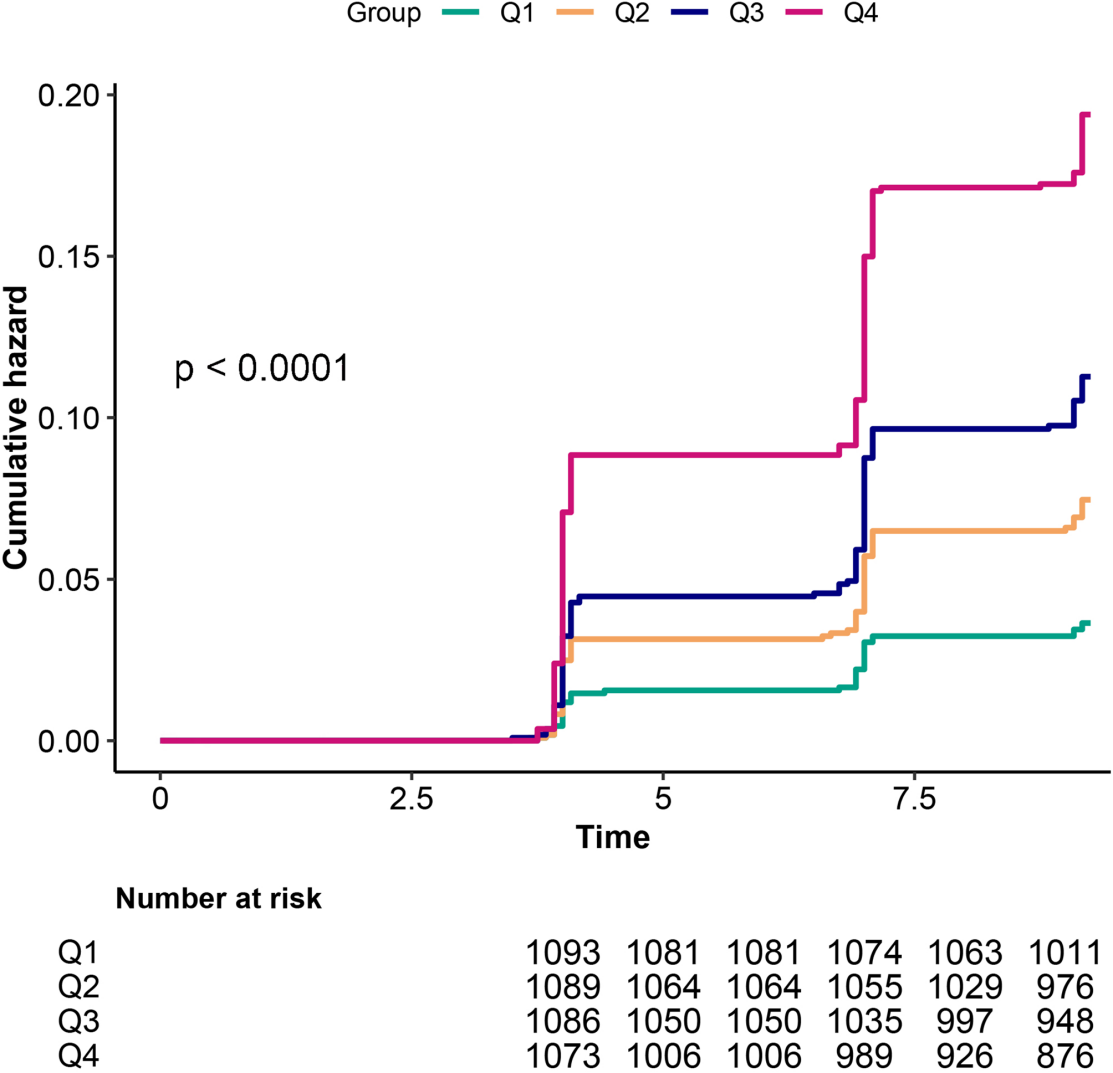
Kaplan-Meier curves for CMM risk stratified by TyG-WHtR index categories

**Fig. 3:**
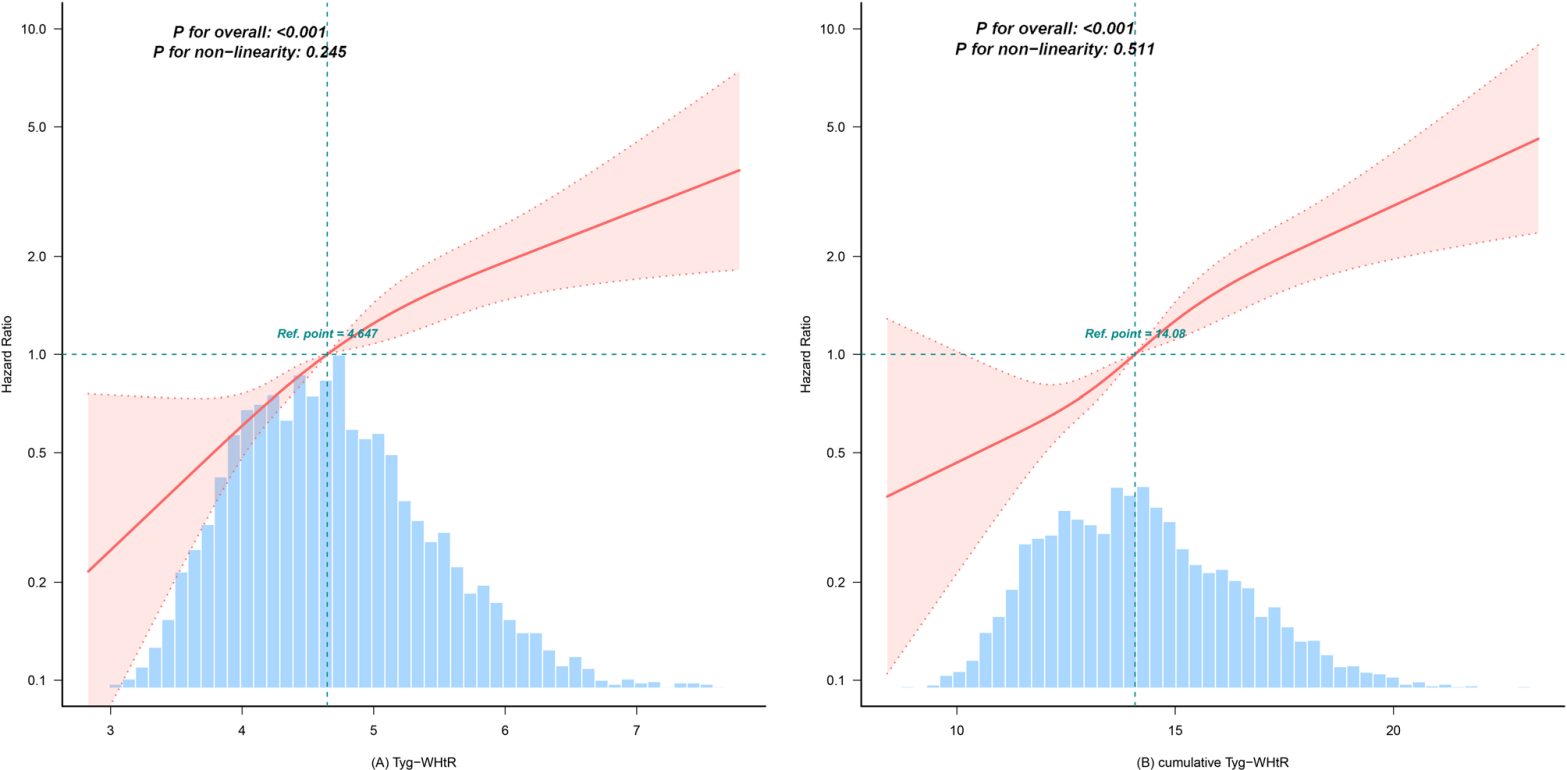
Restricted cubic spline curves for CMM by TyG-WHtR (**A**) and cumulative TyG-WHtR (**B**) after covariate adjustment. Heavy central line represents the estimated adjusted hazard ratio, with shaded ribbons denoting 95% confidence interval. The model is adjuste d for age, gender, marital status, educational level, residence, smoking status, drinking status, hypertension, dyslipidemia, cancer, systolic blood pressure, diastolic blood pressure, total cholesterol, HDL-C, and LDL-C

During a median follow-up of 9 years, 413 (9.40%) of the participants developed CMM. In accordance with TyG-WHtR quartile classification, the occurrence rates of CMM from Q1 to Q4 were 38.45, 76.91, 114.34, and 188.04 per 10,000 person-years, correspondingly. As shown in Fig. 2, the Kaplan-Meier curves illustrate that the cumulative CMM hazard increased progressively from TyG-WHtR Q1 to Q4, and the differences were statistically significant (Fig. 2 log-rank test *P* < 0.0001). For every 1-SD increase in TyG-WHtR, the risk of CMM increased as follows in the different models: (Model 1) 104% (HR = 2.04, 95% CI 1.81–2.29), (Model 2) 103% (HR = 2.03, 95% CI 1.8–2.28), (Model 3) 72% (HR = 1.72, 95% CI 1.52–1.95), (Model 4) 71% (HR = 1.71, 95% CI 1.45–2.01). In the fully adjusted Model 4, progressively higher CMM risks were observed with increasing quartiles relative to TyG-WHtR Q1. The adjusted HRs (95% CI) were 1.75 (1.18–2.6) for Q2, 2.33 (1.58–3.43) for Q3, and 3.13 (2.08–4.7) for Q4. Multivariable-adjusted RCS revealed a linear correlation between TyG-WHtR and CMM, as well as between cumulative TyG-WHtR and CMM (Fig. 3).

Higher cumulative TyG-WHtR levels independently correlated with increased CMM risk. After adjusting for all covariates, a 1-SD increase in the TyG-WHtR index was associated with a 21% higher risk of overall CMM (HR = 1.21, 95% CI 1.15–1.28). Compared to Q1, the HR (95% CI) for CMM in Q2-Q4 were: 1.62 (1.09–2.42), 2.35 (1.59–3.47), and 3.28 (2.17–4.95), respectively. Table S4 (Additional file 1) presents the logistic regression analysis results for heart disease, diabetes, and stroke (Table 2).

**Table 2:** Cox regression analysis for the association between TyG-WHtR and CMM

	Model 1		Model 2		Model 3		Model 4	
	HR (95%CI)	*P* value	HR (95%CI)	*P* value	HR (95%CI)	*P* value	HR (95%CI)	*P* value
TyG-WHtR (per 1 SD)	2.04 (1.81–2.29)	< 0.001	2.03 (1.8–2.28)	< 0.001	1.72 (1.52–1.95)	< 0.001	1.71 (1.45–2.01)	< 0.001
TyG-WHtR quartile								
1	1(Ref)		1(Ref)		1(Ref)		1(Ref)	
2	2.05 (1.39–3.04)	< 0.001	2.05 (1.38–3.02)	< 0.001	1.86 (1.26–2.75)	0.002	1.75 (1.18–2.6)	0.005
3	3.12 (2.15–4.53)	< 0.001	3.08 (2.12–4.47)	< 0.001	2.6 (1.79–3.79)	< 0.001	2.33 (1.58–3.43)	< 0.001
4	5.22 (3.64–7.47)	< 0.001	5.15 (3.59–7.38)	< 0.001	3.7 (2.56–5.34)	< 0.001	3.13 (2.08–4.7)	< 0.001
Trend.test		< 0.001		< 0.001		< 0.001		< 0.001
Cumulative TyG-WHtR (per 1 SD)	1.29(1.25–1.36)	< 0.001	1.3(1.25–1.36)	< 0.001	1.23 (1.17–1.28)	< 0.001	1.21 (1.15–1.28)	< 0.001
Cumulative TyG-WHtR quartile								
1	1(Ref)		1(Ref)		1(Ref)		1(Ref)	
2	1.94 (1.31–2.89)	0.001	1.94 (1.3–2.88)	0.001	1.71 (1.15–2.55)	0.008	1.62 (1.09–2.42)	0.018
3	3.21 (2.21–4.67)	< 0.001	3.17 (2.18–4.6)	< 0.001	2.61 (1.79–3.8)	< 0.001	2.35 (1.59–3.47)	< 0.001
4	5.49 (3.83–7.88)	< 0.001	5.43 (3.78–7.8)	< 0.001	3.85 (2.65–5.58)	< 0.001	3.28 (2.17–4.95)	< 0.001
Trend.test		< 0.001		< 0.001		< 0.001		< 0.001

Model 1 adjusted for age and gender. Model 2 adjusted for age, gender, marital status, educational level, residence, smoking status, and drinking status. Model 3 adjusted for variables in Model 2 and history of hypertension, dyslipdemia, and cancer. Model 4 adjusted for variables in Model 3 and SBP, DBP, TC, HDL-C, and LDL-C

HR, hazard ratio; CI, confidence interval; Ref, reference

### Predictive value of TyG-WHtR for incident CMM

**Fig. 4:**
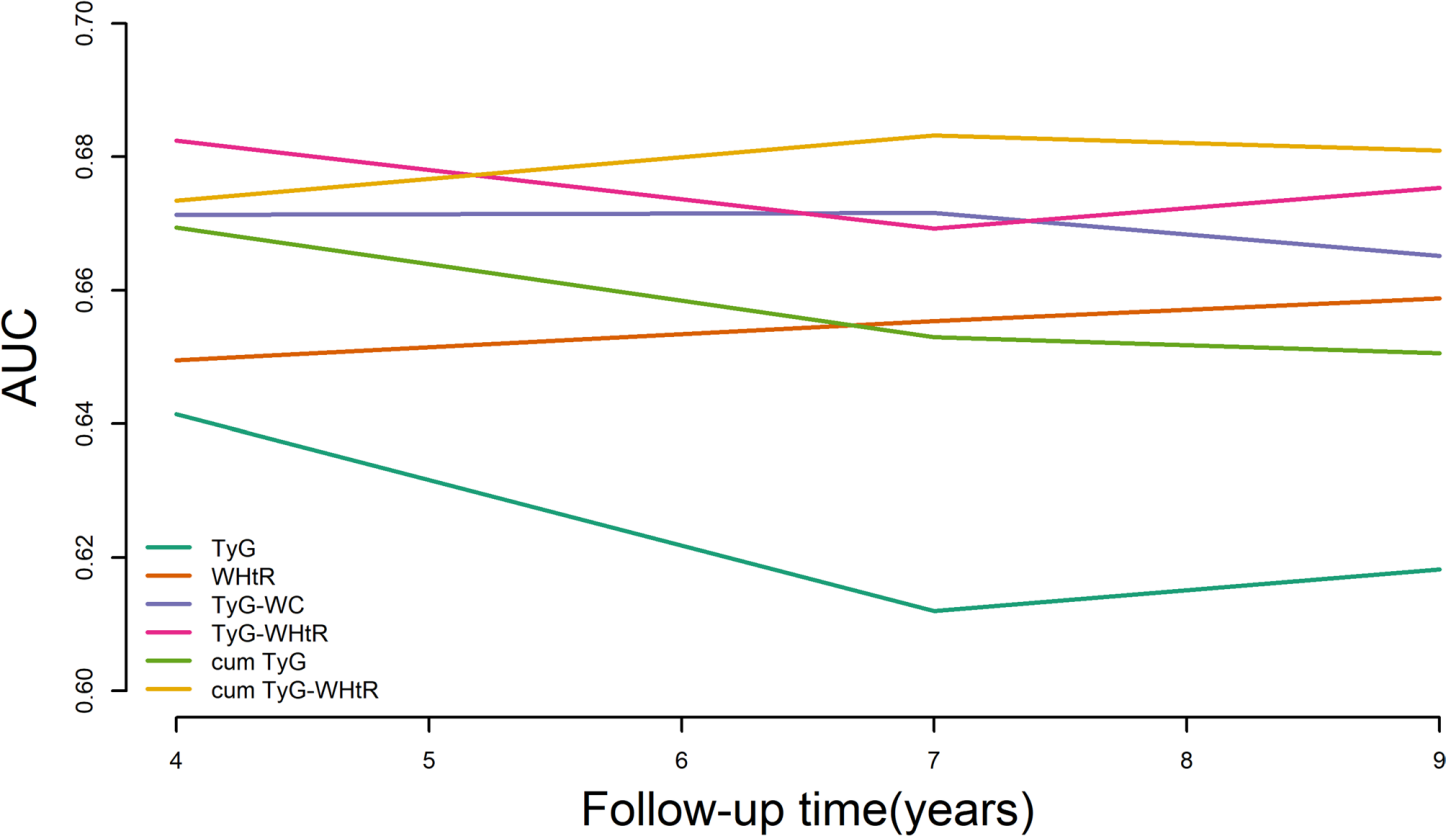
Time-dependent predictive capacity of TyG-WHtR for CMM. AUC, area under the curve; CMM, cardiometabolic multimorbidity; TyG, triglyceride-glucose; WHtR, waist height ratio; TyG-WHtR, triglyceride glucose-waist height ratio index; cum TyG-WHtR, cumulative triglyceride glucose-waist height ratio index; TyG-WC, glucose triglyceride-waist circumference

The predictive value of adding TyG, WHtR, TyG-WC, baseline TyG-WHtR, and cumulative TyG-WHtR to Model 4 for CMM was assessed. Time-dependent ROC analysis (Fig. 4) showed that TyG-WHtR had the highest discriminative ability at year 4 (AUC: 0.682, 95% CI 0.610–0.755), followed by a decline at year 7 (AUC: 0.669, 95% CI 0.634–0.704) and a slight increase at year 9 (AUC: 0.675, 95% CI 0.648–0.703). In contrast, cumulative TyG-WHtR showed a lower AUC at year 4 (AUC: 0.673, 95% CI 0.599–0.748) than TyG-WHtR, but outperformed other indices at year 7 (AUC: 0.683, 95% CI 0.648–0.718) and year 9 (AUC: 0.681, 95% CI 0.653–0.709). Consistent with these findings, Table 3 quantifies the incremental utility using IDI and NRI values. Specifically, both baseline and cumulative TyG-WHtR significantly improved reclassification: for baseline, NRI = 0.101 (95% CI 0.015–0.139) and IDI = 0.010 (95% CI 0.003–0.021); for cumulative, NRI = 0.127 (95% CI 0.032–0.164) and IDI = 0.013 (95% CI 0.005–0.025) (all *P* < 0.05).

**Table 3:** Incremental predictive value of tyg, whtr, TyG-WHtR and cumulative TyG-WHtR for CMM

Model	NRI (95% CI)	*P* value	IDI (95% CI)	*P* value
Basic model	Ref	Ref	Ref	Ref
+TyG	0.061 (0.007–0.111)	0.043	0.004 (-0.001-0.012)	0.092
+TyG-WC	0.014 (0.009–0.134)	0.697	0.001 (0.000-0.002)	0.076
+WHtR	0.076 (0.003–0.118)	0.009	0.007 (0.001–0.016)	0.004
+cumulative TyG	0.078 (0.020–0.151)	0.018	0.010 (0.003–0.021)	< 0.001
+TyG-WHtR	0.101 (0.015–0.139)	0.001	0.010 (0.003–0.021)	< 0.001
+cumulative TyG-WHtR	0.127 (0.032–0.164)	< 0.001	0.013 (0.005–0.025)	< 0.001

The basic model included age, gender, marital status, educational level, smoking status, drinking status, hypertension, dyslipidemia, cancer, systolic blood pressure, diastolic blood pressure, total cholesterol, HDL-C, and LDL-C

NRI, net reclassification improvement; Ref, reference; IDI, integrated discrimination improvement; CI, confidence interval; Ref, reference; TyG, triglyceride glucose; WHtR, waist height ratio; TyG-WHtR, triglyceride glucose-waist height ratio index

### Subgroup analyses

**Fig. 5:**
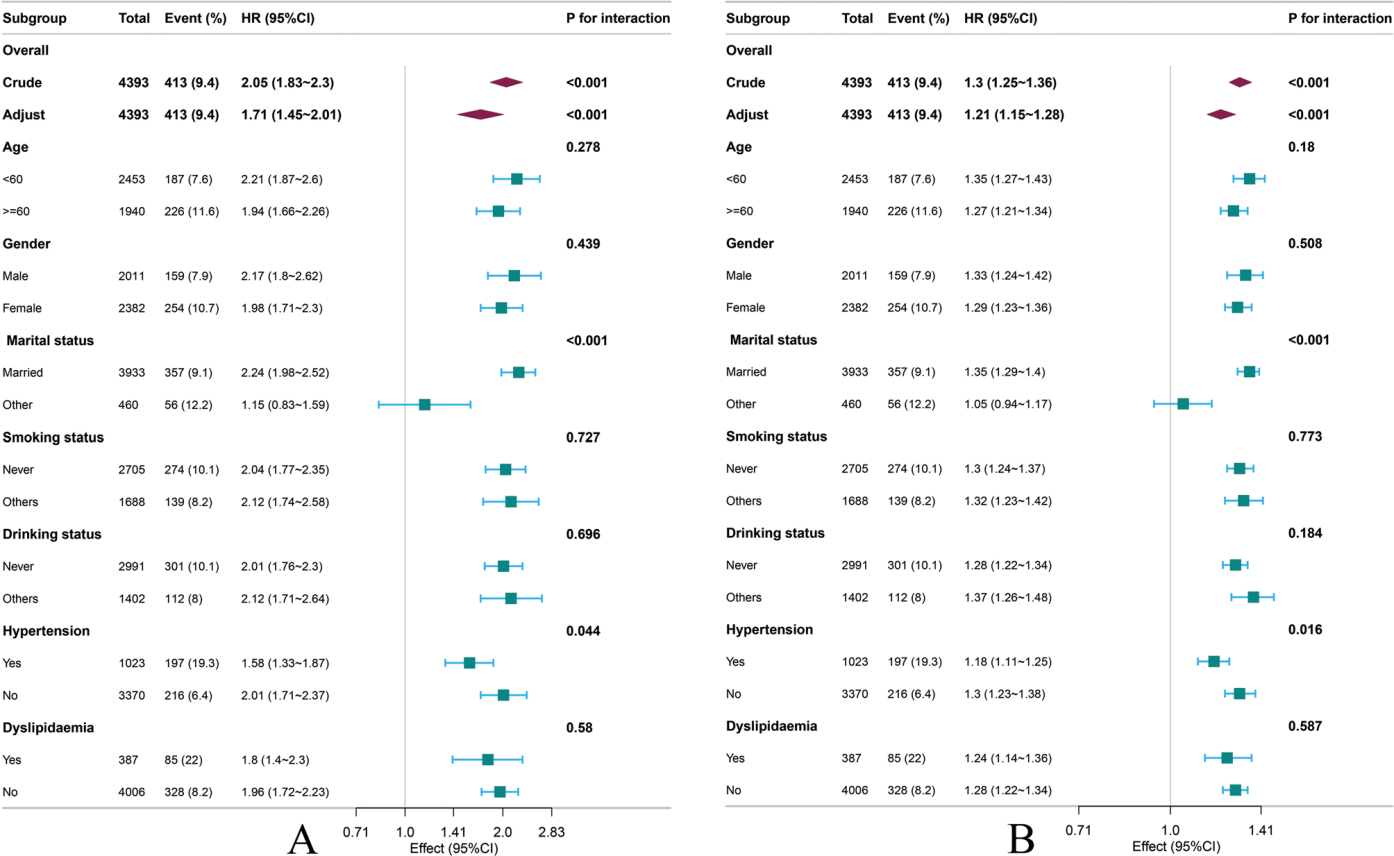
Subgroup analysis of the association between** A** TyG-WHtR,** B** cumulative TyG-WHtR and CMM

Figure 5 shows the associations of TyG-WHtR and cumulative TyG-WHtR with CMM risk after stratification by age, gender, marital status, smoking status, drinking status, hypertension, and dyslipidemia. The stratified analysis revealed significant interaction effects for hypertension and marital status, while no significant interaction effects were observed in the other subgroups (P for interaction > 0.05).

### Sensitivity analyses

To assess the robustness of the key findings, we performed a series of sensitivity analyses. First, consistent results were observed in both the complete-case analysis and the multiple imputation datasets (Supplementary Table S3). Second, after excluding individuals with diabetes, heart disease, or stroke at baseline, TyG-WHtR retained a significant association with CMM during follow-up (Supplementary Table S4). Third, to minimize residual confounding further, we excluded participants with baseline hypertension, dyslipidemia, or cancer. The association remained statistically robust (Supplementary Table S5). Fourth, we observed similar associations after accounting for the competing risks of mortality (Supplementary Figure S1).

## Discussion

Our study comprehensively explored the connection between the TyG-WHtR index and CMM, yielding the following key findings. First, during the 9-year follow-up period, both higher cumulative and baseline TyG-WHtR indices were significantly associated with an increased incidence of CMM, independent of traditional risk factors such as age, sex, and LDL-C levels. Second, a linear relationship was observed between both forms of the TyG-WHtR index and CMM incidence. It is noteworthy that the cumulative TyG-WHtR index showed slightly lower predictive ability than baseline TyG-WHtR in the early follow-up, but outperformed it at later time points. Overall, both indices demonstrated superior predictive performance compared with TyG or WHtR alone. Finally, subgroup analyses revealed that marital status and hypertension significantly modified the associations between the TyG-WHtR indices and CMM. These findings suggest that the TyG-WHtR index plays an important role in identifying individuals at risk of CMM. It could be used as a valuable cardiometabolic risk assessment tool in future public health practice.

Numerous previous studies have focused on the role of the TyG index in predicting individual diseases, including stroke [31], heart failure [32]and diabetes [33]. Chen et al. used the TyG index to predict revascularization after percutaneous coronary intervention in patients with T2DM [34]. Wanlu Su et al. conducted a cohort study in a Chinese population and found that the TyG index was able to identify co-morbidities of type 2 diabetes(T2DM) and hypertension, especially in older adults [35]. Given the limited accuracy and stability of individual biomarkers in risk prediction, recent studies have increasingly focused on combined indices, such as TyG with obesity measures, for improved assessment of chronic disease risk. A prospective cohort study in the UK further validated the reliability of composite indices (TyG-BMI, TyG-WC) in identifying individuals at high risk for stroke [36]. A recent study including 11,937 adults from the National Health and Nutrition Examination Survey (NHANES) compared TyG with related composite indices for cardiovascular disease and found that the TyG-WHtR was superior to the TyG index in terms of diagnostic accuracy [24]. In the general population, TyG-WHtR has been found to be a better predictor of mortality than TyG [37]. Notably, no studies have evaluated the predictive value of TyG-WHtR for CMM risk, as previous studies have focused on individual CMDs. In the present study, we demonstrated for the first time in a large national prospective cohort study that higher TyG-WHtR was significantly associated with increased CMM risk.

Considering that CMM develops over a prolonged period, a single measurement of the TyG index may not be suitable for real-time monitoring and risk stratification during hospitalization [38]. Many studies have attempted to introduce a long-term trajectory and a cumulative TyG index for a more accurate assessment of changes in metabolic status over time, which has been shown to improve the prediction of cardiovascular events [39, 40]. The time-dependent AUC allows for the assessment of the predictive performance of TyG-related indices across different follow-up periods. In our study, cumulative TyG-WHtR demonstrated gradually stronger discrimination for cardiovascular events during longer follow-up. In contrast, baseline TyG-WHtR demonstrated greater predictive ability for cardiovascular risk in the early follow-up period and, given its simplicity, could be considered for use in public health screening programs. A combination of short- and long-term indices could also be an alternative. In addition, the results of NRI and IDI further indicate that both the TyG-WHtR index and the cumulative TyG-WHtR index improve the predictive ability of the baseline model more effectively than other TyG-related indices.

In our study, we apply TyG-WHtR as an early screening tool for CMM, thereby extending the use of TyG-based combined indices in the context of comorbidity. Our results showed a 2.66-fold increase in CMM incidence in the highest quartile compared with the lowest TyG-WHtR quartile. Similarly, results grouped according to cumulative TyG-WHtR quartiles showed similar findings. Patients with higher cumulative TyG-WHtR had a higher risk of CMM. To explore the stability of TyG-WHtR in different groups, we performed subgroup analyses. Interestingly, the association of TyG-WHtR index with CMM was not significant in unmarried and hypertensive populations. On the one hand, unmarried populations are at higher risk of vascular events and all-cause mortality, which may be mediated by underlying nonmetabolic factors, such as social status and economic status [41, 42]. On the other hand, as an independent risk factor for CMM, hypertension interacts with metabolic dysregulation represented by TyG-WHtR, potentially modifying its predictive value. A recent study using mean arterial pressure in combination with the TyG-WHtR index to monitor the risk of CVD had a predictive value that exceeded that of the TyG-WHtR alone [28].In conclusion, the interplay between TyG-WHtR and other factors requires further investigation to fully understand its predictive capacity for CMM in different subgroups.

The pathological association between TyG-WHtR and CMM remains unclear, and potential mechanisms may involve several pathways. IR and visceral fat accumulation promote and interact with each other, and together they increase the risk of CMM through multiple pathologic mechanisms. As visceral adiposity accumulates and undergoes pathological remodeling, adipose tissue releases free fatty acids, pro-inflammatory cytokines, and adipokines, contributing to chronic low-grade inflammation and systemic metabolic disturbances [43–45]. Low-grade inflammation is strongly linked to endothelial dysfunction and atherosclerosis, with an associated increase in cardiovascular risk [46]. Importantly, obesity-induced oxidative stress and inflammatory responses disrupt normal insulin receptor function, impairing insulin signaling and leading to IR [43, 47]. IR is a major risk factor for CMDs, occupying a central position in the metabolic dysfunction network [48–50]. It contributes to impaired glucose metabolism and is closely associated with dyslipidemia and lipotoxicity. This further aggravates metabolic dysfunction, thereby forming a vicious cycle. Research by John R. Petrie has shown that obesity and IR are prevalent in individuals with coexisting type 2 diabetes (T2DM) and hypertension, a condition associated with an increased risk of subsequent cardiovascular events [51]. Many studies have revealed the crosstalk between metabolic and vascular systems, suggesting its role as a pathophysiological mechanism in metabolism-related multimorbidities [52–54].

This study has several strengths. (1) It employed a prospective cohort design using data from CHARLS, with a large sample size and up to 9 years of follow-up. (2) The use of a composite indicator captured the core components of metabolic dysfunction within the comorbidity network, taking into account both disease interactions and shared pathological mechanisms. (3) By comparing baseline and cumulative data, the study comprehensively evaluated the performance of the index, enhancing the robustness and clinical applicability of the findings. However, our study has some limitations: (1) As an observational study, it was not possible to clarify the causal relationship between TyG-WHtR and CMM, and the conclusions obtained were limited to an association interpretation. (2) Although we adjusted for a variety of known confounders (e.g., age, sex, smoking status), potential residual confounders remained. (3) The identification of CMMs was largely based on self-reported data collected during baseline and follow-up surveys, which may introduce information bias. However, it is worth noting that previous studies have shown that self-reported disease diagnoses were found to be in good agreement with medical records. (4) The CHARLS database was developed for the middle-aged and elderly population in the Chinese region. The ethnic and regional limitations of the sample may limit the extrapolation of the findings to other ethnic groups and age groups.

## Conclusion

In this study, we found a significant association between TyG-WHtR and CMM incidence in Chinese middle-aged and elderly people. Specifically, higher levels of TyG-WHtR were strongly associated with an increased risk of CMM, suggesting that this index may be a useful tool for predicting the risk of CMM in the Chinese middle-aged and elderly population. Given that TyG-WHtR is easy to calculate and does not require complex equipment, it can be used as a simple and effective screening tool in clinical practice to help identify individuals who are at higher risk of CMM, thereby facilitating the application of early intervention and prevention strategies.

## Supplementary Information

Below is the link to the electronic supplementary material.

Supplementary Material 1

## Data Availability

No datasets were generated or analysed during the current study.

## Abbreviations

CMM: Cardiometabolic multimorbidity
TyG-WHtR: Triglyceride glucose waist height ratio index
CHARLS: Chinese health and retirement longitudinal study
CMDs: Cardiometabolic diseases
IR: Insulin resistance
HOMA-IR: Homeostatic model assessment of insulin resistance
WC: Waist circumference
WHR: Waist to hip ratio
SBP: Systolic blood pressure
DBP: Diastolic blood pressure
HDL-C: Highdensity lipoprotein cholesterol
LDL-C: Lowdensity lipoprotein cholesterol
TC: Total cholesterol
TG: Triglyceride
HbA1c: Glycosylated hemoglobin
SD: Standard deviation
IQR: Interquartile range
HR: Hazard ratio
CI: Confidence interval
RCS: Restrictedcubic spline
K-M: Kaplan-Meier curve
ROC: Receiveroperating characteristic
AUC: Area under the curve
