# Supplementary material for: The association between triglyceride glucose-waist height ratio index and cardiometabolic multimorbidity among Chinese middle-aged and older adults: a national prospective cohort study
Source: Cardiovasc Diabetol. 2025 Sep 2;24:358. doi: 10.1186/s12933-025-02919-x (PMC12406543; doi:10.1186/s12933-025-02919-x)
Supplement: Supplementary file 1 — Supplementary Material 1 [file 12933_2025_2919_MOESM1_ESM.docx]

**Table S1.** Distribution of missing data.

| Characteristics | No. of missing values | Percent (%) |
| --- | --- | --- |
| Age | 0 | 0 |
| Gender | 3 | 0.06 |
| Marital status | 0 | 0 |
| Educational level | 1 | 0.02 |
| Smoking status | 1 | 0.02 |
| Drinking status | 0 | 0 |
| Hypertension | 23 | 0.52 |
| Dyslipidemia | 76 | 1.73 |
| Cancer | 18 | 0.41 |
| SBP | 26 | 0.59 |
| DBP | 26 | 0.59 |
| HDL_C | 0 | 0 |
| LDL_C | 11 | 0.25 |
| TC | 0 | 0 |

**Table S2**. Collinearity Statistics.

| Variable | GVIF | DF | GVIF^(1/(2*Df)) |
| --- | --- | --- | --- |
| TyG-WHtR | 2.013 | 1 | 1.419 |
| Age | 1.423 | 1 | 1.193 |
| Gender | 2.553 | 1 | 1.598 |
| Marital status | 1.175 | 1 | 1.084 |
| Educational level | 1.457 | 2 | 1.099 |
| Residence | 1.081 | 1 | 1.04 |
| Smoking status | 2.057 | 1 | 1.434 |
| Drinking status | 1.406 | 1 | 1.186 |
| Hypertension | 1.26 | 1 | 1.122 |
| Dyslipidemia | 1.159 | 1 | 1.076 |
| Cancer | 1.011 | 1 | 1.006 |
| SBP | 1.961 | 1 | 1.4 |
| DBP | 1.929 | 1 | 1.389 |
| HDL-C | 1.449 | 1 | 1.204 |
| LDL-C | 3.111 | 1 | 1.764 |
| TC | 3.515 | 1 | 1.875 |

**Table S3.** Baseline characteristics of participants by cumulative TyG-WHtR

| **Characteristic** | **Total**  **(n = 4393)** | cumulative **TyG-WHtR** | | | | **P** |
| --- | --- | --- | --- | --- | --- | --- |
|  |  | **Q1 (n = 1098)** | **Q2 (n = 1098)** | **Q3 (n = 1098)** | **Q4 (n = 1099)** |  |
| Age, years (Mean ± SD) | 58.76 ± 8.70 | 59.30 ± 9.02 | 58.90 ± 8.73 | 58.06 ± 8.45 | 58.80 ± 8.53 | 0.009 |
| Gender, n(%) |  |  |  |  |  | < 0.001 |
| Male | 2011 (45.8) | 737 (67.1) | 547 (49.8) | 439 (40) | 288 (26.2) |  |
| Female | 2382 (54.2) | 361 (32.9) | 551 (50.2) | 659 (60) | 811 (73.8) |  |
| Marital status, n(%) |  |  |  |  |  | 0.191 |
| Married | 3933 (89.5) | 987 (89.9) | 970 (88.3) | 999 (91) | 977 (88.9) |  |
| Others | 460 (10.5) | 111 (10.1) | 128 (11.7) | 99 (9) | 122 (11.1) |  |
| Educational level, n(%) |  |  |  |  |  | < 0.001 |
| Illiteracy | 1177 (26.8) | 260 (23.7) | 290 (26.4) | 278 (25.3) | 349 (31.8) |  |
| Middle school below | 1910 (43.5) | 497 (45.3) | 498 (45.4) | 456 (41.5) | 459 (41.8) |  |
| Middle school and above | 1306 (29.7) | 341 (31.1) | 310 (28.2) | 364 (33.2) | 291 (26.5) |  |
| Residence, n(%) |  |  |  |  |  | < 0.001 |
| Rural | 3813 (86.8) | 1022 (93.1) | 960 (87.4) | 918 (83.6) | 913 (83.1) |  |
| Urban | 580 (13.2) | 76 (6.9) | 138 (12.6) | 180 (16.4) | 186 (16.9) |  |
| Smoking status, n(%) |  |  |  |  |  | < 0.001 |
| Never | 2705 (61.6) | 476 (43.4) | 651 (59.3) | 725 (66) | 853 (77.6) |  |
| Others | 1688 (38.4) | 622 (56.6) | 447 (40.7) | 373 (34) | 246 (22.4) |  |
| Drinking status, n(%) |  |  |  |  |  | < 0.001 |
| Current | 1402 (31.9) | 470 (42.8) | 373 (34) | 320 (29.1) | 239 (21.7) |  |
| Others | 2991 (68.1) | 628 (57.2) | 725 (66) | 778 (70.9) | 860 (78.3) |  |
| Comorbidities, n(%) |  |  |  |  |  |  |
| Hypertension | 1023 (23.3) | 122 (11.1) | 208 (18.9) | 281 (25.6) | 412 (37.5) | < 0.001 |
| Dyslipidemia | 387 ( 8.8) | 37 (3.4) | 69 (6.3) | 108 (9.8) | 173 (15.7) | < 0.001 |
| Cancer | 36 ( 0.8) | 6 (0.5) | 8 (0.7) | 8 (0.7) | 14 (1.3) | 0.259 |
| SBP,mmHg（Mean ± SD） | 130.53 ± 23.65 | 124.14 ± 19.22 | 128.79 ± 23.49 | 130.51 ± 20.15 | 138.67 ± 28.31 | < 0.001 |
| DBP,mmHg（Mean ± SD） | 76.16 ± 12.21 | 72.40 ± 11.57 | 75.13 ± 12.02 | 76.51 ± 11.34 | 80.60 ± 12.45 | < 0.001 |
| HDL-C,mg/dL（Mean ± SD） | 51.18 ± 15.62 | 59.49 ± 16.61 | 53.63 ± 14.20 | 49.10 ± 13.56 | 42.52 ± 12.69 | < 0.001 |
| LDL-C,mg/dL（Mean ± SD） | 116.24 ± 35.46 | 109.10 ± 31.17 | 115.23 ± 31.91 | 122.01 ± 35.00 | 118.62 ± 41.57 | < 0.001 |
| TC,mg/dL（Mean ± SD） | 193.41 ± 39.20 | 182.82 ± 35.76 | 187.88 ± 36.28 | 197.16 ± 36.31 | 205.76 ± 43.89 | < 0.001 |
| HbA1C, % | 5.23 ± 0.75 | 5.10 ± 0.56 | 5.14 ± 0.63 | 5.21 ± 0.66 | 5.49 ± 1.01 | < 0.001 |
| TG2012,mg/dL | 105.3 (74.3, 153.1) | 73.5 (57.5, 95.6) | 93.8 (71.7, 128.3) | 114.2 (85.0, 160.0) | 166.4 (117.7, 249.1) | < 0.001 |
| TG2015,mg/dL | 115.0 (83.2, 168.1) | 79.6 (64.6, 100.9) | 100.9 (78.8, 134.5) | 129.6 (97.3, 174.3) | 183.2 (137.2, 270.8) | < 0.001 |
| FBG2012,mg/dL | 109.26 ± 34.10 | 100.64 ± 18.52 | 105.62 ± 31.53 | 107.80 ± 27.03 | 122.97 ± 48.04 | < 0.001 |
| FBG2015,mg/dL | 102.58 ± 32.69 | 92.85 ± 20.08 | 97.82 ± 22.88 | 102.00 ± 28.33 | 117.66 ± 46.93 | < 0.001 |
| Height2012（Mean ± SD） | 157.69 ± 8.35 | 160.26 ± 7.92 | 157.79 ± 8.31 | 157.36 ± 8.37 | 155.36 ± 8.05 | < 0.001 |
| WC2012（Mean ± SD） | 85.24 ± 9.95 | 75.55 ± 5.98 | 81.96 ± 6.32 | 87.74 ± 6.40 | 95.71 ± 7.70 | < 0.001 |
| Height2015（Mean ± SD） | 157.74 ± 23.45 | 161.29 ± 36.44 | 157.29 ± 8.39 | 156.85 ± 8.65 | 155.53 ± 26.62 | < 0.001 |
| WC2015（Mean ± SD） | 86.26 ± 17.29 | 75.29 ± 6.50 | 82.74 ± 6.31 | 89.26 ± 6.74 | 97.73 ± 28.20 | < 0.001 |
| TyG2012（Mean ± SD） | 8.68 ± 0.67 | 8.22 ± 0.44 | 8.51 ± 0.48 | 8.75 ± 0.55 | 9.25 ± 0.71 | < 0.001 |
| TyG2015（Mean ± SD） | 8.71 ± 0.63 | 8.23 ± 0.40 | 8.53 ± 0.45 | 8.81 ± 0.49 | 9.29 ± 0.61 | < 0.001 |
| WHtR2012（Mean ± SD） | 0.54 ± 0.07 | 0.47 ± 0.03 | 0.52 ± 0.03 | 0.56 ± 0.04 | 0.62 ± 0.05 | < 0.001 |
| WHtR2015（Mean ± SD） | 0.55 ± 0.07 | 0.47 ± 0.04 | 0.53 ± 0.03 | 0.57 ± 0.04 | 0.63 ± 0.05 | < 0.001 |
| TyG-WHtR2012（Mean ± SD） | 4.71 ± 0.76 | 3.87 ± 0.30 | 4.42 ± 0.29 | 4.87 ± 0.30 | 5.69 ± 0.52 | < 0.001 |
| TyG-WHtR2015（Mean ± SD） | 4.79 ± 0.81 | 3.87 ± 0.34 | 4.48 ± 0.30 | 5.01 ± 0.31 | 5.82 ± 0.52 | < 0.001 |
| Heart disease, n (%) | 1257 (28.6) | 240 (21.9) | 275 (25) | 345 (31.4) | 397 (36.1) | < 0.001 |
| Diabetes, n (%) | 1169 (26.6) | 143 (13) | 212 (19.3) | 308 (28.1) | 506 (46) | < 0.001 |
| Stroke, n (%) | 461 (10.5) | 73 (6.6) | 116 (10.6) | 111 (10.1) | 161 (14.6) | < 0.001 |
| CMM, n (%) | 413 ( 9.4) | 38 (3.5) | 72 (6.6) | 113 (10.3) | 190 (17.3) | < 0.001 |

Except for variables with time labels, all other variables are from Wave 1.

SBP: systolic blood pressure; DBP: diastolic blood pressure; TC: total cholesterol; TG: triglycerides; HDL-C: high-density lipoprotein cholesterol; LDL-C: low-density lipoprotein cholesterol; HbA1C: glycated hemoglobin; FBG: fasting blood glucose; WC: waist measurements; TyG: triglyceride-glucose; WHtR: waist height ratio; TyG-WHtR: triglyceride glucose-waist height ratio index; Q: quartile

**Table S4.** The results of logistic regression analyses for CVD, Diabetes and Stroke.

| **CVD** | | | | | | | | |
| --- | --- | --- | --- | --- | --- | --- | --- | --- |
|  | Model 1 | | Model 2 | | Model 3 | | Model 4 | |
|  | HR (95%CI) | P value | HR (95%CI) | P value | HR (95%CI) | P value | HR (95%CI) | P value |
| TyG-WHtR (per 1 SD) | 1.32 (1.21~1.45) | <0.001 | 1.33 (1.21~1.45) | <0.001 | 1.12 (1.02~1.23) | 0.018 | 1.17 (1.04~1.33) | 0.011 |
| TyG-WHtR quartile | | | | | | | | |
| 1 | 1(Ref) |  | 1(Ref) |  | 1(Ref) |  | 1(Ref) |  |
| 2 | 1.2 (0.98~1.46) | 0.078 | 1.2 (0.98~1.47) | 0.071 | 1.11 (0.9~1.36) | 0.324 | 1.11 (0.9~1.36) | 0.327 |
| 3 | 1.57 (1.3~1.92) | <0.001 | 1.58 (1.29~1.92) | <0.001 | 1.36 (1.11~1.66) | 0.003 | 1.37 (1.1~1.69) | 0.004 |
| 4 | 1.74 (1.43~2.12) | <0.001 | 1.75 (1.43~2.13) | <0.001 | 1.29 (1.04~1.58) | 0.018 | 1.35 (1.05~1.72) | 0.018 |
| Trend.test |  | <0.001 |  | <0.001 |  | 0.005 |  | 0.005 |
| Cumulative TyG-WHtR (per 1 SD) | 1.11 (1.08~1.15) | <0.001 | 1.11 (1.08~1.15) | <0.001 | 1.05 (1.02~1.09) | 0.004 | 1.06 (1.02~1.11) | 0.004 |
| Cumulative TyG-WHtR quartile | | | | | | | | |
| 1 | 1(Ref) |  | 1(Ref) |  | 1(Ref) |  | 1(Ref) |  |
| 2 | 1.15 (0.94~1.4) | 0.18 | 1.16 (0.95~1.42) | 0.156 | 1.04 (0.85~1.28) | 0.691 | 1.04 (0.85~1.29) | 0.682 |
| 3 | 1.57 (1.29~1.91) | <0.001 | 1.57 (1.29~1.92) | <0.001 | 1.31 (1.07~1.61) | 0.009 | 1.32 (1.07~1.64) | 0.011 |
| 4 | 1.82 (1.49~2.22) | <0.001 | 1.83 (1.5~2.24) | <0.001 | 1.32 (1.07~1.64) | 0.009 | 1.39 (1.09~1.78) | 0.009 |
| Trend.test |  | <0.001 |  | <0.001 |  | 0.002 |  | 0.002 |
| **Diabetes** | | | | | | | | |
|  | Model 1 | | Model 2 | | Model 3 | | Model 4 | |
|  | HR (95%CI) | P value | HR (95%CI) | P value | HR (95%CI) | P value | HR (95%CI) | P value |
| TyG-WHtR (per 1 SD) | 2.36 (2.14~2.6) | <0.001 | 2.36 (2.14~2.6) | <0.001 | 2.22 (2.01~2.46) | <0.001 | 2.21 (1.94~2.51) | <0.001 |
| TyG-WHtR quartile | | | | | | | | |
| 1 | 1(Ref) |  | 1(Ref) |  | 1(Ref) |  | 1(Ref) |  |
| 2 | 1.54 (1.23~1.93) | <0.001 | 1.49 (1.19~1.86) | <0.001 | 1.49 (1.19~1.86) | <0.001 | 1.41 (1.12~1.78) | 0.003 |
| 3 | 2.35 (1.89~2.92) | <0.001 | 2.21 (1.78~2.74) | <0.001 | 2.21 (1.78~2.74) | <0.001 | 1.98 (1.57~2.5) | <0.001 |
| 4 | 5.16 (4.16~6.4) | <0.001 | 4.75 (3.87~5.84) | <0.001 | 4.75 (3.87~5.84) | <0.001 | 3.84 (2.97~4.97) | <0.001 |
| Trend.test |  | <0.001 |  | <0.001 |  | <0.001 |  | <0.001 |
| Cumulative TyG-WHtR (per 1 SD) | 1.4 (1.35~1.45) | <0.001 | 1.4 (1.35~1.45) | <0.001 | 1.37 (1.32~1.42) | <0.001 | 1.37 (1.31~1.43) | <0.001 |
| Cumulative TyG-WHtR quartile | | | | | | | | |
| 1 | 1(Ref) |  | 1(Ref) |  | 1(Ref) |  | 1(Ref) |  |
| 2 | 1.69 (1.34~2.14) | <0.001 | 1.69 (1.34~2.14) | <0.001 | 1.62 (1.28~2.05) | <0.001 | 1.59 (1.25~2.02) | <0.001 |
| 3 | 2.89 (2.31~3.62) | <0.001 | 2.9 (2.31~3.63) | <0.001 | 2.7 (2.15~3.39) | <0.001 | 2.56 (2.01~3.26) | <0.001 |
| 4 | 6.54 (5.23~8.17) | <0.001 | 6.55 (5.23~8.2) | <0.001 | 5.81 (4.61~7.31) | <0.001 | 5.27 (4.04~6.86) | <0.001 |
| Trend.test |  | <0.001 |  | <0.001 |  | <0.001 |  | <0.001 |
| **Stroke** | | | | | | | | |
|  | Model 1 | | Model 2 | | Model 3 | | Model 4 | |
|  | HR (95%CI) | P value | HR (95%CI) | P value | HR (95%CI) | P value | HR (95%CI) | P value |
| TyG-WHtR (per 1 SD) | 1.49 (1.32~1.7) | <0.001 | 1.5 (1.32~1.7) | <0.001 | 1.31 (1.15~1.5) | <0.001 | 1.26 (1.06~1.5) | 0.009 |
| TyG-WHtR quartile | | | | | | | | |
| 1 | 1(Ref) |  | 1(Ref) |  | 1(Ref) |  | 1(Ref) |  |
| 2 | 1.43 (1.05~1.95) | 0.024 | 1.44 (1.06~1.97) | 0.021 | 1.35 (0.99~1.85) | 0.058 | 1.29 (0.94~1.77) | 0.122 |
| 3 | 1.82 (1.35~2.46) | <0.001 | 1.83 (1.35~2.48) | <0.001 | 1.62 (1.19~2.21) | 0.002 | 1.48 (1.07~2.04) | 0.019 |
| 4 | 2.36 (1.75~3.18) | <0.001 | 2.37 (1.76~3.21) | <0.001 | 1.86 (1.36~2.55) | <0.001 | 1.61 (1.12~2.32) | 0.01 |
| Trend.test |  | <0.001 |  | <0.001 |  | <0.001 |  | 0.009 |
| Cumulative TyG-WHtR (per 1 SD) | 1.15 (1.1~1.2) | <0.001 | 1.15 (1.1~1.2) | <0.001 | 1.1 (1.04~1.15) | <0.001 | 1.07 (1.01~1.13) | 0.024 |
| Cumulative TyG-WHtR quartile | | | | | | | | |
| 1 | 1(Ref) |  | 1(Ref) |  | 1(Ref) |  | 1(Ref) |  |
| 2 | 1.66 (1.22~2.25) | 0.001 | 1.75 (1.28~2.38) | <0.001 | 1.62 (1.18~2.21) | 0.003 | 1.52 (1.11~2.09) | 0.009 |
| 3 | 1.58 (1.16~2.15) | 0.004 | 1.74 (1.26~2.38) | 0.001 | 1.5 (1.09~2.07) | 0.014 | 1.37 (0.97~1.92) | 0.07 |
| 4 | 2.41 (1.8~3.22) | <0.001 | 2.7 (1.98~3.67) | <0.001 | 2.09 (1.52~2.88) | <0.001 | 1.8 (1.25~2.6) | 0.002 |
| Trend.test |  | <0.001 |  | <0.001 |  | <0.001 |  | 0.008 |

Model 1 adjusted for age and gender. Model 2 adjusted for age, gender, marital status, educational level，residence, smoking status, and drinking status. Model 3 adjusted for variables in Model 2 and history of hypertension, dyslipdemia, and cancer. Model 4 adjusted for variables in Model 3 and SBP, DBP, TC, HDL-C, and LDL-C.

HR: hazard ratio; CI: confidence interval; Ref: reference.

**Table S5** Sensitivity analyses of the TyG-WHtR on the risks of developing CMM in terms of data missing

|  | Model 1 | | Model 2 | | Model 3 | | Model 4 | |
| --- | --- | --- | --- | --- | --- | --- | --- | --- |
|  | HR (95%CI) | P value | HR (95%CI) | P value | HR (95%CI) | P value | HR (95%CI) | P value |
| TyG-WHtR  (per 1 SD) | 2.04 (1.81~2.29) | <0.001 | 1.99 (1.71~2.32) | <0.001 | 1.7 (1.45~2) | <0.001 | 1.68 (1.36~2.07) | <0.001 |
| TyG-WHtR quartile | | | | | | | | |
| 1 | 1(Ref) |  | 1(Ref) |  | 1(Ref) |  | 1(Ref) |  |
| 2 | 2.06 (1.39~3.04) | <0.001 | 2 (1.26~3.17) | 0.003 | 1.82 (1.14~2.91) | 0.012 | 1.66 (1.03~2.67) | 0.037 |
| 3 | 3.12 (2.15~4.52) | <0.001 | 2.92 (1.87~4.54) | <0.001 | 2.53 (1.61~3.97) | <0.001 | 2.16 (1.34~3.46) | 0.001 |
| 4 | 5.21 (3.64~7.46) | <0.001 | 4.55 (2.96~6.99) | <0.001 | 3.37 (2.17~5.25) | <0.001 | 2.89 (1.75~4.79) | <0.001 |
| Trend.test |  | <0.001 |  | <0.001 |  | <0.001 |  | <0.001 |
| Cumulative TyG-WHtR (per 1 SD) | 1.3 (1.25~1.36) | <0.001 | 1.28 (1.21~1.35) | <0.001 | 1.21 (1.14~1.28) | <0.001 | 1.19 (1.11~1.27) | <0.001 |
| Cumulative TyG-WHtR quartile | | | | | | | | |
| 1 | 1(Ref) |  | 1(Ref) |  | 1(Ref) |  | 1(Ref) |  |
| 2 | 1.95 (1.31~2.89) | 0.001 | 1.85 (1.16~2.93) | 0.009 | 1.63 (1.02~2.62) | 0.041 | 1.49 (0.93~2.41) | 0.1 |
| 3 | 3.22 (2.22~4.67) | <0.001 | 2.85 (1.84~4.42) | <0.001 | 2.42 (1.54~3.79) | <0.001 | 2.07 (1.3~3.32) | 0.002 |
| 4 | 5.49 (3.83~7.87) | <0.001 | 4.76 (3.11~7.29) | <0.001 | 3.5 (2.25~5.45) | <0.001 | 2.93 (1.78~4.83) | <0.001 |
| Trend.test |  | <0.001 |  | <0.001 |  | <0.001 |  | <0.001 |

Model 1 adjusted for age and gender. Model 2 adjusted for age, gender, marital status, educational level，residence, smoking status, and drinking status. Model 3 adjusted for variables in Model 2 and history of hypertension, dyslipdemia, and cancer. Model 4 adjusted for variables in Model 3 and SBP, DBP, TC, HDL-C, and LDL-C.

HR: hazard ratio; CI: confidence interval; Ref: reference.

**Table S6** Sensitivity analysis of the TyG-WHtR on the risks of developing CMM in participants without diabetes, heart disease or stroke at baseline (N=3,646)

|  | Model 1 | | Model 2 | | Model 3 | | Model 4 | |
| --- | --- | --- | --- | --- | --- | --- | --- | --- |
|  | HR (95%CI) | P value | HR (95%CI) | P value | HR (95%CI) | P value | HR (95%CI) | P value |
| TyG-WHtR (per 1 SD) | 1.9 (1.6~2.26) | <0.001 | 1.91 (1.61~2.27) | <0.001 | 1.74 (1.45~2.09) | <0.001 | 1.82 (1.44~2.3) | <0.001 |
|  |  |  |  |  |  |  |  |  |
| TyG-WHtR quartile | | | | | | | | |
| 1 | 1(Ref) |  | 1(Ref) |  | 1(Ref) |  | 1(Ref) |  |
| 2 | 1.43 (0.87~2.36) | 0.158 | 1.44 (0.88~2.38) | 0.149 | 1.37 (0.83~2.26) | 0.217 | 1.28 (0.77~2.12) | 0.337 |
| 3 | 2.65 (1.69~4.17) | <0.001 | 2.69 (1.71~4.24) | <0.001 | 2.47 (1.56~3.91) | <0.001 | 2.19 (1.36~3.54) | 0.001 |
| 4 | 3.7 (2.36~5.81) | <0.001 | 3.77 (2.4~5.92) | <0.001 | 3.15 (1.99~5) | <0.001 | 2.84 (1.68~4.8) | <0.001 |
| Trend.test | 1.57 (1.37~1.79) | <0.001 | 1.58 (1.38~1.8) | <0.001 | 1.49 (1.3~1.71) | <0.001 | 1.45 (1.23~1.7) | <0.001 |
| Cumulative TyG-WHtR (per 1 SD) | 1.26 (1.19~1.34) | <0.001 | 1.27 (1.19~1.34) | <0.001 | 1.23 (1.15~1.3) | <0.001 | 1.22 (1.13~1.31) | <0.001 |
| Cumulative TyG-WHtR quartile | | | | | | | | |
| 1 | 1(Ref) |  | 1(Ref) |  | 1(Ref) |  | 1(Ref) |  |
| 2 | 1.89 (1.12~3.19) | 0.017 | 1.92 (1.14~3.24) | 0.015 | 1.81 (1.07~3.06) | 0.027 | 1.68 (0.99~2.86) | 0.054 |
| 3 | 3.48 (2.14~5.67) | <0.001 | 3.55 (2.18~5.79) | <0.001 | 3.23 (1.98~5.29) | <0.001 | 2.88 (1.73~4.81) | <0.001 |
| 4 | 4.6 (2.82~7.48) | <0.001 | 4.69 (2.88~7.65) | <0.001 | 3.92 (2.38~6.47) | <0.001 | 3.41 (1.96~5.95) | <0.001 |
| Trend.test | 1.63 (1.42~1.86) | <0.001 | 1.63 (1.43~1.87) | <0.001 | 1.54 (1.34~1.77) | <0.001 | 1.49 (1.26~1.75) | <0.001 |

Model 1 adjusted for age and gender. Model 2 adjusted for age, gender, marital status, educational level，residence, smoking status, and drinking status. Model 3 adjusted for variables in Model 2 and history of hypertension, dyslipdemia, and cancer. Model 4 adjusted for variables in Model 3 and SBP, DBP, TC, HDL-C, and LDL-C.

HR: hazard ratio; CI: confidence interval; Ref: reference.

**Table S7** Sensitivity analyses of the TyG-WHtR on the risks of developing CMM in participants without chronic disease (hypertension, dyslipidemia, cancer, diabetes, heart disease or stroke) at baseline.(N=2,770)

|  | Model 1 | | Model 2 | | Model 3 | | Model 4 | |
| --- | --- | --- | --- | --- | --- | --- | --- | --- |
|  | HR (95%CI) | P value | HR (95%CI) | P value | HR (95%CI) | P value | HR (95%CI) | P value |
| TyG-WHtR  (per 1 SD) | 2 (1.6~2.52) | <0.001 | 2.03 (1.62~2.56) | <0.001 | 1.92 (1.42~2.61) | <0.001 | 1.92 (1.42~2.61) | <0.001 |
| TyG-WHtR quartile | | | | | | | | |
| 1 | 1(Ref) |  | 1(Ref) |  | 1(Ref) |  | 1(Ref) |  |
| 2 | 1.47 (0.82~2.65) | 0.196 | 1.51 (0.84~2.72) | 0.166 | 1.3 (0.72~2.36) | 0.389 | 1.3 (0.72~2.36) | 0.389 |
| 3 | 2.47 (1.43~4.27) | 0.001 | 2.57 (1.49~4.45) | 0.001 | 2.01 (1.12~3.6) | 0.019 | 2.01 (1.12~3.6) | 0.019 |
| 4 | 3.89 (2.26~6.72) | <0.001 | 4.02 (2.33~6.96) | <0.001 | 3.03 (1.6~5.74) | 0.001 | 3.03 (1.6~5.74) | 0.001 |
| Trend.test | 1.59 (1.34~1.88) | <0.001 | 1.6 (1.36~1.9) | <0.001 | 1.47 (1.2~1.8) | <0.001 | 1.47 (1.2~1.8) | <0.001 |
| Cumulative TyG-WHtR (per 1 SD) | 1.29 (1.19~1.4) | <0.001 | 1.3 (1.2~1.41) | <0.001 | 1.25 (1.13~1.38) | <0.001 | 1.25 (1.13~1.38) | <0.001 |
| Cumulative TyG-WHtR quartile | | | | | | | | |
| 1 | 1(Ref) |  | 1(Ref) |  | 1(Ref) |  | 1(Ref) |  |
| 2 | 1.96 (1.07~3.58) | 0.028 | 2.01 (1.1~3.67) | 0.023 | 1.73 (0.94~3.18) | 0.08 | 1.73 (0.94~3.18) | 0.08 |
| 3 | 3.03 (1.7~5.42) | <0.001 | 3.18 (1.78~5.7) | <0.001 | 2.55 (1.39~4.69) | 0.003 | 2.55 (1.39~4.69) | 0.003 |
| 4 | 4.73 (2.66~8.43) | <0.001 | 4.91 (2.75~8.78) | <0.001 | 3.52 (1.82~6.81) | <0.001 | 3.52 (1.82~6.81) | <0.001 |
| Trend.test | 1.64 (1.38~1.94) | <0.001 | 1.66 (1.4~1.96) | <0.001 | 1.5 (1.23~1.83) | <0.001 | 1.5 (1.23~1.83) | <0.001 |

Model 1 adjusted for age and gender. Model 2 adjusted for age, gender, marital status, educational level，residence, smoking status, and drinking status. Model 3 adjusted for variables in Model 2 and history of hypertension, dyslipdemia, and cancer. Model 4 adjusted for variables in Model 3 and SBP, DBP, TC, HDL-C, and LDL-C.

HR: hazard ratio; CI: confidence interval; Ref: reference.

**Table S8** Association of TyG-WHtR with the risk of CMM stratified by different factors

| Subgroup | Variable | n.total | n.event(%) | HR (95%CI) | P value | P for interaction |
| --- | --- | --- | --- | --- | --- | --- |
| Age |  |  |  |  |  | 0.022 |
| <60 |  |  |  |  |  |  |
|  | Q1 | 592 | 16 (2.7) | 1(Ref) |  |  |
|  | Q2 | 624 | 24 (3.8) | 1.17 (0.62~2.22) | 0.63 |  |
|  | Q3 | 635 | 62 (9.8) | 2.49 (1.4~4.44) | 0.002 |  |
|  | Q4 | 602 | 85 (14.1) | 2.82 (1.52~5.26) | 0.001 |  |
| >=60 |  |  |  |  |  |  |
|  | Q1 | 506 | 22 (4.3) | 1(Ref) |  |  |
|  | Q2 | 474 | 52 (11) | 2.3 (1.39~3.83) | 0.001 |  |
|  | Q3 | 463 | 51 (11) | 2.04 (1.2~3.46) | 0.008 |  |
|  | Q4 | 497 | 101 (20.3) | 3.25 (1.88~5.62) | <0.001 |  |
| Gender |  |  |  |  |  | 0.487 |
| Male |  |  |  |  |  |  |
|  | Q1 | 707 | 22 (3.1) | 1(Ref) |  |  |
|  | Q2 | 548 | 43 (7.8) | 2 (1.18~3.37) | 0.01 |  |
|  | Q3 | 457 | 44 (9.6) | 1.97 (1.14~3.4) | 0.015 |  |
|  | Q4 | 299 | 50 (16.7) | 2.82 (1.56~5.11) | 0.001 |  |
| Female |  |  |  |  |  |  |
|  | Q1 | 391 | 16 (4.1) | 1(Ref) |  |  |
|  | Q2 | 550 | 33 (6) | 1.41 (0.77~2.57) | 0.268 |  |
|  | Q3 | 641 | 69 (10.8) | 2.4 (1.37~4.21) | 0.002 |  |
|  | Q4 | 800 | 136 (17) | 3.25 (1.83~5.75) | <0.001 |  |
| Marital status |  |  |  |  |  | 0.001 |
| Married |  |  |  |  |  |  |
|  | Q1 | 978 | 27 (2.8) | 1(Ref) |  |  |
|  | Q2 | 988 | 66 (6.7) | 2.12 (1.34~3.33) | 0.001 |  |
|  | Q3 | 992 | 93 (9.4) | 2.59 (1.65~4.05) | <0.001 |  |
|  | Q4 | 975 | 171 (17.5) | 3.92 (2.46~6.24) | <0.001 |  |
| Other |  |  |  |  |  |  |
|  | Q1 | 120 | 11 (9.2) | 1(Ref) |  |  |
|  | Q2 | 110 | 10 (9.1) | 0.88 (0.36~2.17) | 0.79 |  |
|  | Q3 | 106 | 20 (18.9) | 1.9 (0.8~4.54) | 0.146 |  |
|  | Q4 | 124 | 15 (12.1) | 0.99 (0.35~2.8) | 0.979 |  |
| Smoking status |  |  |  |  |  | 0.796 |
| Other |  |  |  |  |  |  |
|  | Q1 | 602 | 24 (4) | 1(Ref) |  |  |
|  | Q2 | 443 | 32 (7.2) | 1.57 (0.91~2.68) | 0.102 |  |
|  | Q3 | 379 | 39 (10.3) | 1.78 (1.03~3.07) | 0.04 |  |
|  | Q4 | 264 | 44 (16.7) | 2.43 (1.33~4.45) | 0.004 |  |
| Never |  |  |  |  |  |  |
|  | Q1 | 496 | 14 (2.8) | 1(Ref) |  |  |
|  | Q2 | 655 | 44 (6.7) | 2.1 (1.14~3.85) | 0.017 |  |
|  | Q3 | 719 | 74 (10.3) | 3.1 (1.72~5.59) | <0.001 |  |
|  | Q4 | 835 | 142 (17) | 4.14 (2.27~7.56) | <0.001 |  |
| Drinking status |  |  |  |  |  | 0.677 |
| Other |  |  |  |  |  |  |
|  | Q1 | 455 | 12 (2.6) | 1(Ref) |  |  |
|  | Q2 | 376 | 26 (6.9) | 2.2 (1.1~4.41) | 0.027 |  |
|  | Q3 | 322 | 32 (9.9) | 2.81 (1.39~5.68) | 0.004 |  |
|  | Q4 | 249 | 42 (16.9) | 4.49 (2.11~9.59) | <0.001 |  |
| Never |  |  |  |  |  |  |
|  | Q1 | 643 | 26 (4) | 1(Ref) |  |  |
|  | Q2 | 722 | 50 (6.9) | 1.56 (0.96~2.52) | 0.072 |  |
|  | Q3 | 776 | 81 (10.4) | 2.13 (1.34~3.38) | 0.001 |  |
|  | Q4 | 850 | 144 (16.9) | 2.77 (1.71~4.49) | <0.001 |  |
| Hypertension |  |  |  |  |  | 0.18 |
| No |  |  |  |  |  |  |
|  | Q1 | 963 | 24 (2.5) | 1(Ref) |  |  |
|  | Q2 | 899 | 50 (5.6) | 2.16 (1.31~3.55) | 0.002 |  |
|  | Q3 | 817 | 57 (7) | 2.61 (1.57~4.34) | <0.001 |  |
|  | Q4 | 691 | 85 (12.3) | 4.62 (2.69~7.93) | <0.001 |  |
| Yes |  |  |  |  |  |  |
|  | Q1 | 135 | 14 (10.4) | 1(Ref) |  |  |
|  | Q2 | 199 | 26 (13.1) | 1.18 (0.61~2.28) | 0.628 |  |
|  | Q3 | 281 | 56 (19.9) | 1.73 (0.94~3.18) | 0.077 |  |
|  | Q4 | 408 | 101 (24.8) | 1.78 (0.95~3.33) | 0.073 |  |
| Dyslipidaemia |  |  |  |  |  | 0.719 |
| No |  |  |  |  |  |  |
|  | Q1 | 1057 | 34 (3.2) | 1(Ref) |  |  |
|  | Q2 | 1022 | 68 (6.7) | 1.86 (1.22~2.83) | 0.004 |  |
|  | Q3 | 1005 | 95 (9.5) | 2.36 (1.56~3.57) | <0.001 |  |
|  | Q4 | 922 | 131 (14.2) | 3.2 (2.05~4.97) | <0.001 |  |
| Yes |  |  |  |  |  |  |
|  | Q1 | 41 | 4 (9.8) | 1(Ref) |  |  |
|  | Q2 | 76 | 8 (10.5) | 0.9 (0.27~3.07) | 0.871 |  |
|  | Q3 | 93 | 18 (19.4) | 1.57 (0.5~4.92) | 0.438 |  |
|  | Q4 | 177 | 55 (31.1) | 2.11 (0.67~6.59) | 0.199 |  |

TyG-WHtR: triglyceride glucose-waist height ratio; CMM: cardiometabolic multimorbidity; HR: hazard ratio; CI: confidence interval; Ref: reference.

**Table S9** Association of cumulative TyG-WHtR with the risk of CMM stratified by different factors

| Subgroup | Variable | n.total | n.event(%) | HR (95%CI) | P value | P for interaction |
| --- | --- | --- | --- | --- | --- | --- |
| Age |  |  |  |  |  | 0.187 |
| <60 |  |  |  |  |  |  |
|  | Q1 | 576 | 14 (2.4) | 1(Ref) |  |  |
|  | Q2 | 612 | 24 (3.9) | 1.34 (0.69~2.61) | 0.394 |  |
|  | Q3 | 646 | 55 (8.5) | 2.53 (1.36~4.68) | 0.003 |  |
|  | Q4 | 619 | 94 (15.2) | 3.69 (1.94~7.05) | <0.001 |  |
| >=60 |  |  |  |  |  |  |
|  | Q1 | 522 | 24 (4.6) | 1(Ref) |  |  |
|  | Q2 | 486 | 48 (9.9) | 1.84 (1.11~3.04) | 0.018 |  |
|  | Q3 | 452 | 58 (12.8) | 2.15 (1.28~3.6) | 0.004 |  |
|  | Q4 | 480 | 96 (20) | 2.85 (1.66~4.91) | <0.001 |  |
| Gender |  |  |  |  |  | 0.27 |
| Male |  |  |  |  |  |  |
|  | Q1 | 737 | 21 (2.8) | 1(Ref) |  |  |
|  | Q2 | 547 | 40 (7.3) | 1.94 (1.13~3.33) | 0.016 |  |
|  | Q3 | 439 | 51 (11.6) | 2.68 (1.55~4.63) | <0.001 |  |
|  | Q4 | 288 | 47 (16.3) | 3.22 (1.76~5.9) | <0.001 |  |
| Female |  |  |  |  |  |  |
|  | Q1 | 361 | 17 (4.7) | 1(Ref) |  |  |
|  | Q2 | 551 | 32 (5.8) | 1.08 (0.59~1.95) | 0.81 |  |
|  | Q3 | 659 | 62 (9.4) | 1.76 (1.01~3.07) | 0.048 |  |
|  | Q4 | 811 | 143 (17.6) | 2.78 (1.58~4.88) | <0.001 |  |
| Marital status |  |  |  |  |  | 0.013 |
| Married |  |  |  |  |  |  |
|  | Q1 | 987 | 27 (2.7) | 1(Ref) | 0.009 |  |
|  | Q2 | 970 | 58 (6) | 1.86 (1.17~2.97) | <0.001 |  |
|  | Q3 | 999 | 101 (10.1) | 2.91 (1.86~4.56) | <0.001 |  |
|  | Q4 | 977 | 171 (17.5) | 4.06 (2.54~6.51) | <0.001 |  |
| Other |  |  |  |  |  |  |
|  | Q1 | 111 | 11 (9.9) | 1(Ref) | 0.639 |  |
|  | Q2 | 128 | 14 (10.9) | 0.81 (0.35~1.92) | 0.941 |  |
|  | Q3 | 99 | 12 (12.1) | 1.04 (0.4~2.69) | 0.628 |  |
|  | Q4 | 122 | 19 (15.6) | 1.27 (0.48~3.35) | 0.484 |  |
| Smoking status |  |  |  |  |  | 0.147 |
| Other |  |  |  |  |  |  |
|  | Q1 | 622 | 18 (2.9) | 1(Ref) | 0.003 |  |
|  | Q2 | 447 | 38 (8.5) | 2.42 (1.36~4.29) | 0.001 |  |
|  | Q3 | 373 | 43 (11.5) | 2.85 (1.57~5.16) | <0.001 |  |
|  | Q4 | 246 | 40 (16.3) | 3.54 (1.83~6.84) | <0.001 |  |
| Never |  |  |  |  |  |  |
|  | Q1 | 476 | 20 (4.2) | 1(Ref) | 0.832 |  |
|  | Q2 | 651 | 34 (5.2) | 1.06 (0.61~1.86) | 0.014 |  |
|  | Q3 | 725 | 70 (9.7) | 1.92 (1.14~3.23) | <0.001 |  |
|  | Q4 | 853 | 150 (17.6) | 2.85 (1.67~4.85) | <0.001 |  |
| Drinking status |  |  |  |  |  | 0.089 |
| Other |  |  |  |  |  |  |
|  | Q1 | 470 | 10 (2.1) | 1(Ref) | 0.023 |  |
|  | Q2 | 373 | 25 (6.7) | 2.39 (1.13~5.05) | <0.001 |  |
|  | Q3 | 320 | 40 (12.5) | 4.53 (2.16~9.47) | <0.001 |  |
|  | Q4 | 239 | 37 (15.5) | 5 (2.25~11.12) | <0.001 |  |
| Never |  |  |  |  |  |  |
|  | Q1 | 628 | 28 (4.5) | 1(Ref) | 0.241 |  |
|  | Q2 | 725 | 47 (6.5) | 1.33 (0.83~2.14) | 0.017 |  |
|  | Q3 | 778 | 73 (9.4) | 1.76 (1.11~2.8) | <0.001 |  |
|  | Q4 | 860 | 153 (17.8) | 2.76 (1.71~4.45) | <0.001 |  |
| Hypertension |  |  |  |  |  | 0.102 |
| No |  |  |  |  |  |  |
|  | Q1 | 976 | 25 (2.6) | 1(Ref) | 0.006 |  |
|  | Q2 | 890 | 47 (5.3) | 2 (1.22~3.28) | <0.001 |  |
|  | Q3 | 817 | 56 (6.9) | 2.59 (1.56~4.3) | <0.001 |  |
|  | Q4 | 687 | 88 (12.8) | 4.69 (2.77~7.95) | <0.001 |  |
| Yes |  |  |  |  |  |  |
|  | Q1 | 122 | 13 (10.7) | 1(Ref) | 0.931 |  |
|  | Q2 | 208 | 25 (12) | 0.97 (0.49~1.92) | 0.122 |  |
|  | Q3 | 281 | 57 (20.3) | 1.65 (0.88~3.1) | 0.115 |  |
|  | Q4 | 412 | 102 (24.8) | 1.7 (0.88~3.28) | 0.024 |  |
| Dyslipidaemia |  |  |  |  |  | 0.933 |
| No |  |  |  |  |  |  |
|  | Q1 | 1061 | 35 (3.3) | 1(Ref) | 0.016 |  |
|  | Q2 | 1029 | 64 (6.2) | 1.68 (1.1~2.56) | <0.001 |  |
|  | Q3 | 990 | 93 (9.4) | 2.33 (1.53~3.53) | <0.001 |  |
|  | Q4 | 926 | 136 (14.7) | 3.33 (2.14~5.17) | <0.001 |  |
| Yes |  |  |  |  |  |  |
|  | Q1 | 37 | 3 (8.1) | 1(Ref) |  |  |
|  | Q2 | 69 | 8 (11.6) | 1.13 (0.29~4.44) | 0.863 |  |
|  | Q3 | 108 | 20 (18.5) | 1.85 (0.52~6.61) | 0.345 |  |
|  | Q4 | 173 | 54 (31.2) | 2.39 (0.66~8.72) | 0.186 |  |

TyG-WHtR: triglyceride glucose-waist height ratio; CMM: cardiometabolic multimorbidity; HR: hazard ratio; CI: confidence interval; Ref: reference.


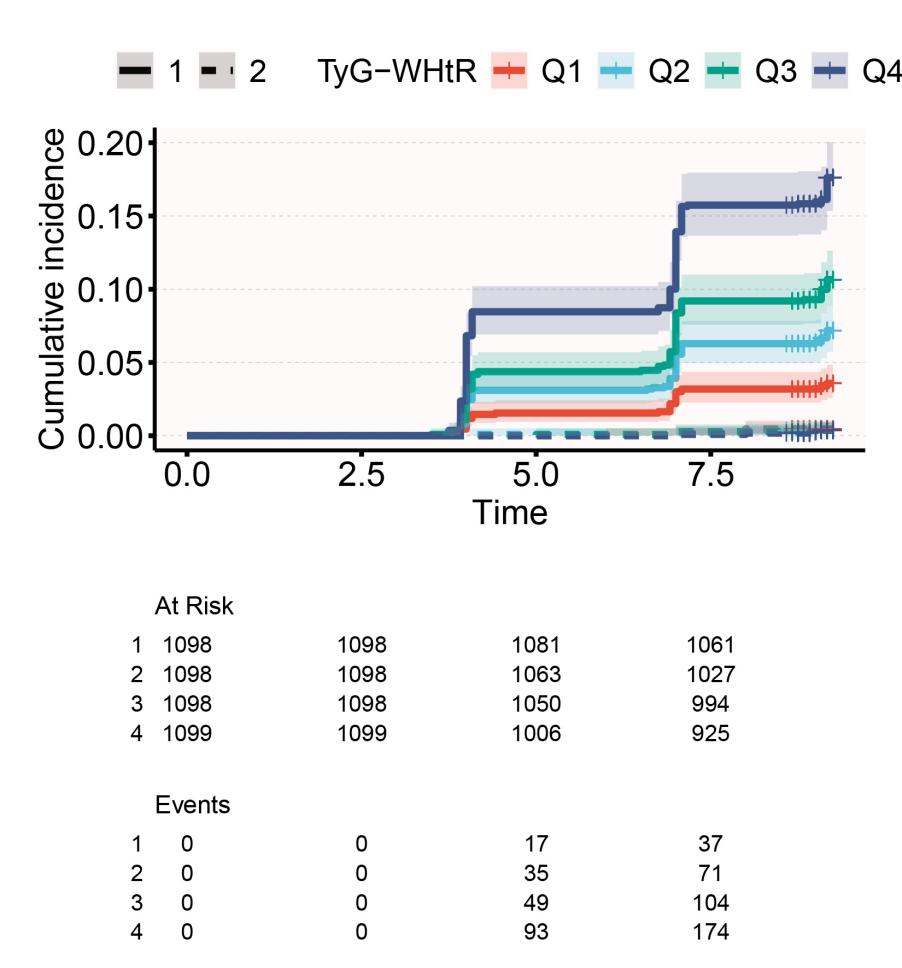


**Figure S1** Cumulative incidence of CMM considering death as a competing risk.The figure depicts the cumulative incidence of cardiometabolic multimorbidity (CMM, solid line) and the competing risk of death from non-CMM causes (dashed line), estimated using the Fine-Gray subdistribution hazards regression model.
